# Light Changes Promote Distinct Responses of Plastid Protein Acetylation Marks

**DOI:** 10.1016/j.mcpro.2024.100845

**Published:** 2024-09-24

**Authors:** Jürgen Eirich, Jean-Baptiste Boyer, Laura Armbruster, Aiste Ivanauskaite, Carolina De La Torre, Thierry Meinnel, Markus Wirtz, Paula Mulo, Iris Finkemeier, Carmela Giglione

**Affiliations:** 1 Plant Physiology, Institute of Plant Biology and Biotechnology, University of Muenster, Muenster, Germany; 2 Université Paris-Saclay, CEA, CNRS, Institute for Integrative Biology of the Cell (I2BC), Gif-sur-Yvette, France; 3 Centre for Organismal Studies Heidelberg, University of Heidelberg, Heidelberg, Germany; 4 Molecular Plant Biology, Department of Life Technologies, University of Turku, Turku, Finland; 5 NGS Core Facility, Medical Faculty Mannheim, University of Heidelberg, Mannheim, Germany

**Keywords:** acetyltransferase, acetylation, quantitative proteomics, light acclimation, *Arabidopsis thaliana*

## Abstract

Protein acetylation is a key co- and post-translational modification. However, how different types of acetylation respond to environmental stress is still unknown. To address this, we investigated the role of a member of the newly discovered family of plastid acetyltransferases (GNAT2), which features both lysine- and N-terminal acetyltransferase activities. Our study aimed to provide a holistic multi-omics acetylation-dependent view of plant acclimation to short-term light changes. We found that both the yield and coverage of the N-terminal acetylome remained unchanged in WT and *gnat2*-KO backgrounds after 2 h of exposure to high light or darkness. Similarly, no differences in transcriptome or adenylate energy charge were observed between the genotypes under the tested light conditions. In contrast, the lysine acetylome proved to be sensitive to the changes in light conditions, especially in the *gnat2* background. This suggests unique strategies of plant acclimation for quick responses to environmental changes involving lysine, but not N-terminal, GNAT2-mediated acetylation activity.

## Highlights

• Comprehensive multi-omics profiling of plant light response.
• Parallel N-terminal and K-acetylation analysis of dual specific GNAT2 and WT.
• N-terminal acetylome remained unchanged after 2 h change in light conditions.
• GNAT2-associated lysine acetylome displayed sensitivity to changes in light.
• Distinct protein acetylation responses to light acclimation.

## Graphical Abstract

**Figure:**
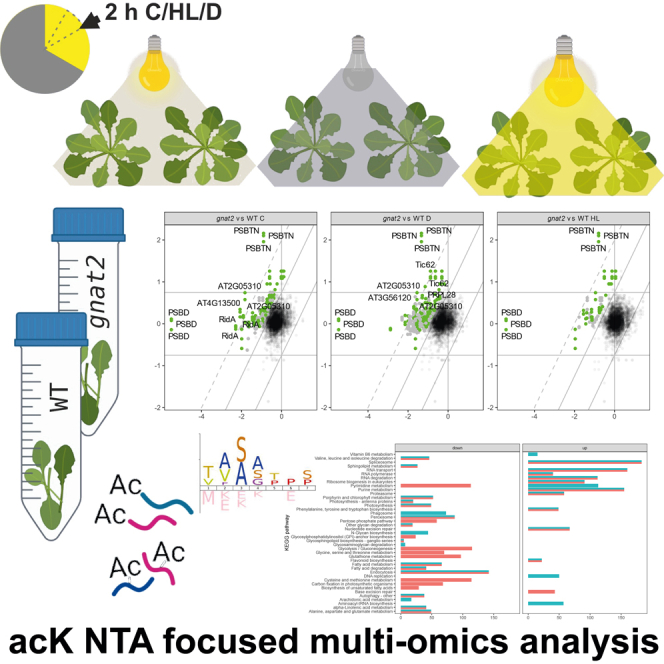

## In Brief

We investigated protein acetylation and the dual-specific plastid acetyltransferase GNAT2 in plant acclimation to short-term light changes. Our multi-omics analysis showed that the N-terminal acetylome remained unchanged in WT and *gnat2*-KO plants after 2 h of high light or darkness. However, the lysine acetylome was sensitive to light conditions, especially in the *gnat2* background. This suggests that plants use distinct acclimation strategies involving GNAT2-mediated lysine acetylation, but not N-terminal acetylation, for rapid environmental responses.

Protein acetylation is the most frequent protein modification after phosphorylation in eukaryotes, including plants (1). N-acetylation refers to the addition of an acetyl moiety on amino groups of lysine side chains (acK) or on acetylation of N-termini (NTA) of proteins through an amide bond. While a majority (>75%) of eukaryotic proteins have their N-terminus entirely modified, not all lysine chains display acK. As proteins have an average of 32 lysines (2), many of them are nevertheless expected to display either one or both acetylation marks, that is, NTA or acK. Acetylation marks have been demonstrated to play crucial roles in all cellular processes. For instance, acK was initially observed on histone proteins where it is involved in the regulation of transcription (3). Since then, proteomic studies have revealed thousands of non-histone proteins containing acK sites (4), where acetylation affects protein functions and can block lysine residues from other protein modifications such as ubiquitination (5). For an increasing number of these proteins, acK was shown to occur in diverse subcellular compartments, including the cytosol and chloroplast, and it was demonstrated that acK can regulate the activity of certain enzymes such as RuBisCO and malate dehydrogenase for example (6, 7, 8). In most cases, acK and NTA are supported by distinct families of acetyltransferases (9). All N-terminal acetyltransferases identified so far belong to the GCN5-family (GNAT) of N-terminal acetyltransferases (NATs), whereas lysine acetyltransferases (KATs) are found in at least three families: GNAT, MYST, and p300/CBP (10). Unlike acK, which occurs post-translationally and is reversibly controlled by specific deacetylases, NTA is regarded as an irreversible modification occurring mostly co-translationally (1). In a few cases, NTA was described to occur post-translationally on hormone peptides or on exported proteins of apicomplexa (11). Only recently it was shown that NTA occurs both co-translationally on plastid-encoded proteins and post-translationally on a significant fraction of nuclear-encoded, plastid-imported proteins after cleavage of the transit peptide (2, 12). A new family of nuclear-encoded acetyltransferases localized in plastids was identified and revealed to display unique features including unexpectedly dual NTA and acK activities (2). Because of their unique mode of action, all these plastid-localized acetyltransferases were named GNATs, and two subfamilies, NAA70 and NAA90 were identified (1). To date, the *in planta* dual activity of the plastid GNATs was demonstrated only for AtGNAT2—one of the three members of the NAA90 subfamily—by showing a global reduction of both plastid NTA and acK in *gnat2* KO plants (2, 13). The protein acetylation activity of GNAT2 was also reported for the rice homolog (14). For rice and *Arabidopsis thaliana*, GNAT2 weak serotonin acetyltransferase (SNAT) activities were reported as well (15, 16). However, the melatonin content of the Arabidopsis KO plants was decreased only upon challenging of the plant with avirulent pathogens or high light (17, 18). Moreover, the *gnat2* plants showed changes in the accumulation of various other compounds, such as ascorbate and Nα-acetyl-L-arginine (19). Interestingly, *gnat2*-KO plants are more susceptible to pathogen infection, which was suggested to depend on its SNAT activity (17). Still, many open questions remain regarding this new GNAT family, such as why there are so many plastid GNATs, why they display dual KA and NTA activities, and what the physiological role of each of them is?

Plants regularly face adverse conditions, such as rapid changes in light intensity, temperature, and water or nutrient availability. Upon perturbation of cellular homeostasis, protein-based signaling networks are activated to orchestrate physiological responses, by regulating gene expression, translation, protein function, and turnover. Signal-responsive protein modifications are emerging as a key mechanism for controlling plant stress responses (20). Protein acetylation is one such modification. Recent studies have shown that protein acetylation in plants is highly dynamic in response to many different stresses, particularly when dealing with cytosolic NATs (21, 22). Loss of *At*GNAT2 causes a defect in the dynamics of photosynthetic light harvesting, and *gnat2* mutants are unable to balance the distribution of excitation energy between the two photosystems (PS) in a process called state transitions (13, 23, 24). While this causes relatively subtle growth defects under standard laboratory growth conditions, the *gnat2* plants show severely retarded growth under fluctuating light (24). Moreover, although both acK and NTA were affected in the *gnat2* plants, the most sensitive acK and NTA targets in these mutant lines were different (4, 13). Several proteins involved in photosynthesis were only affected in their acK status but not in overall abundance, and therefore it is likely that GNAT2-catalyzed acetylation plays a role in the light response. Decreased acetylation levels of specific photosynthesis-related proteins suggested that there may be defects in the interaction of PSI and light harvesting complex II (LHCII), strengthened binding of LHCII to PSII, or more indirect effects on thylakoid macrostructure (13, 23, 24). It has also been suggested that the identified extensive NTA of several LHC proteins might be involved in the membrane folding of grana stacks (25).

Light is one of the major regulatory factors controlling germination, growth, and general fitness of plants, and therefore, they must be able to acclimate to short- and long-term changes in light quality and quantity (26). Here, we performed a multi-omics analysis including deep qualitative and quantitative characterization of N-terminal and lysine acetylation of proteins in the context of WT *versus gnat2* plants to understand the function of GNAT2 in light acclimation. To this end, we treated mature Arabidopsis plants, both WT and *gnat2* mutants, in high light, under standard growth conditions or in darkness for 2 h. The extensive data set revealed that both WT and *gnat2* mutants responded similarly to changes in light conditions, since no major differences in transcriptome, proteome, or energy metabolite composition were revealed between the two genotypes. Further characterization of the impact of these different light conditions revealed that the N-terminal acetylome remained widely unchanged. A strong light-independent reduction on specific plastid proteins was observed in *gnat2* compared to WT. The lysine acetylome proved to be very sensitive to the changes in light conditions especially in the *gnat2* background, suggesting that acK sites change within the investigated timeframe during acclimation, while N-terminal acetylation changes might be associated with long-term responses. Taken together, our data reveals unique strategies of plant acclimation to the different light treatments involving specific PTMs and suggests that plastid lysine and N-terminal acetylations may respond differently to environmental or developmental stimuli.

## Experimental Procedures

### Experimental Design and Statistical Rationale

Experiments were carried out in four replicates. WT plants were used to compare *gnat2* KO plants with. They were treated for 2 h in the dark, under standard growth conditions (as control) or under high light illumination in parallel. For the respective multi-omics experiments, different, well-established analysis pipelines were chosen. As a result, cut-offs for significance were chosen to best fit the respective nature of the data. The analytical and statistical approaches are outlined in detail in the respective method sections below.

### Plant Growth and Light Treatments

**Fig. 1:**
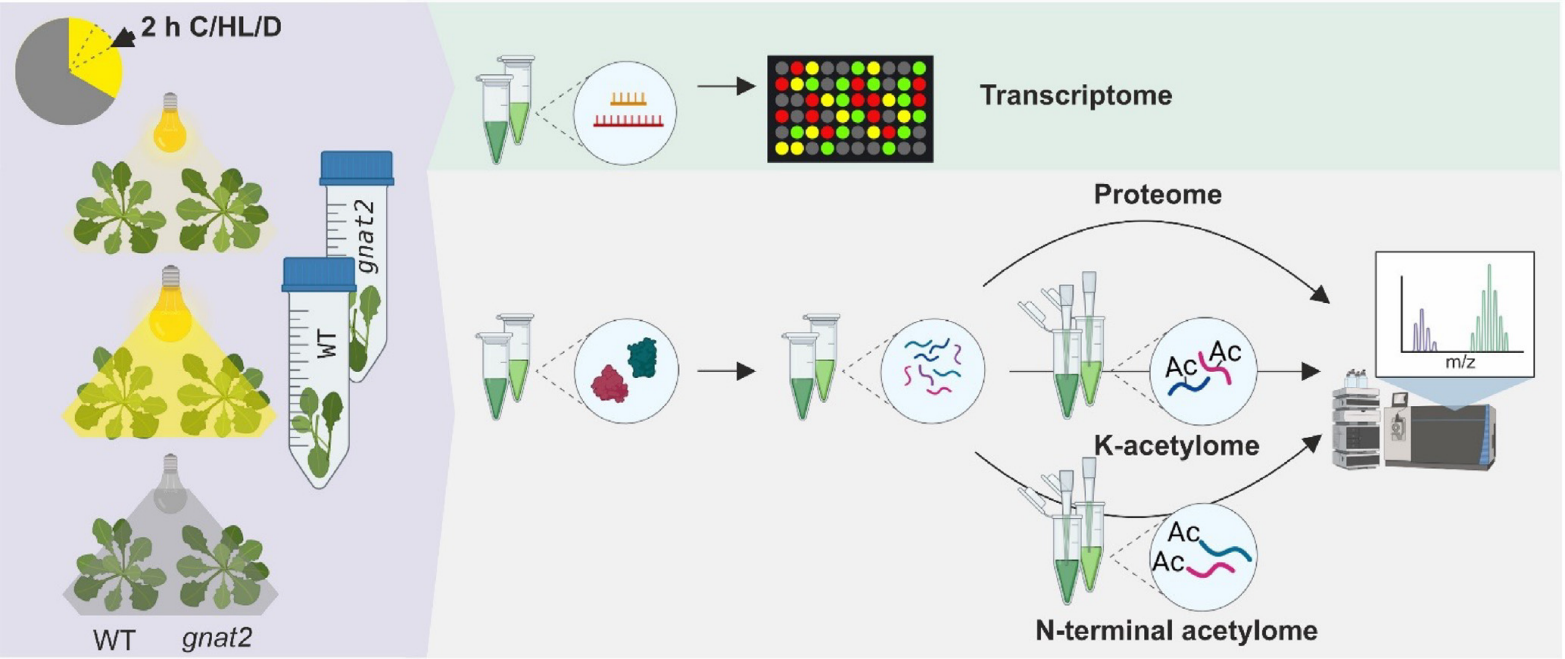
**Scheme of the experimental workflow.** 4.5-week-old *gnat2* and WT plants were grown on soil under short-day conditions (8 h light/16 h dark) at 120 μmol m^-2^s^-1^. After 4.5 weeks, the plants were subjected to different light regimes interrupting the short-day light cycle. Two hours after the regular onset of light, the plants were treated with high light illumination (HL, 1000 μmol m^-2^s^-1^) or darkness (D) for 2 h. The control group (C) continued growth at 120 μmol m^-2^s^-1^. Treatment and sampling time is indicated in the chart. Samples were harvested and flash frozen. RNA, metabolites, and proteins were extracted from leaf tissue for a multi-omics profiling. Modified peptides were enriched with acK-specific antibodies or strong cation exchange chromatography in case of N-terminal acetylation. All experiments were performed on four biological replicates. Icons were created with BioRender.com.

*A. thaliana* ecotype Col-0 and *gnat2* T-DNA KO (SALK_033944) plants were grown for 4.5 weeks in a short day light regime (8 h light/16 h darkness) at PPFD of 120 μmol m^−2^ s^−1^ (light source: Osram Powerstar HQI-BT 400 W/D daylight), 50% humidity, and 23 °C. Two hours after the onset of light, plants were treated for 2 h in the dark (D), under standard growth conditions (GL) or under high light illumination (HL; 1000 μmol m^−2^ s^−1^, 23 °C). Immediately after the treatments, the rosettes were harvested and frozen in liquid nitrogen (Fig. 1). Four distinct biological replicates were assessed. The same material from each treatment was subjected to transcriptome, metabolome, acetylome, and proteomic analyses.

### Transcriptomics

The peqGOLD Total RNA kit (Peqlab) was used to extract RNA from 100 mg plant powder. A global transcriptome analysis was performed using the Affymetrix AraGene-1_0-st arrays (Thermo Fisher Scientific) as described in detail by (21). Biotinylated antisense cDNA was then prepared according to the standard labelling protocol with the GeneChip WT Plus Reagent Kit and the GeneChip Hybridization, Wash and Stain Kit (both from Thermo Fisher Scientific). Afterwards, the hybridization on the chip was performed on a GeneChip Hybridization oven 640, then dyed in the GeneChip Fluidics Station 450, and thereafter scanned with a GeneChip Scanner 3000 (Affymetrix).

Arrays were annotated with a Custom CDF Version 22 with TAIR based gene definitions (27). The raw fluorescence intensity values were normalized applying quantile normalization and RMA background correction. OneWay-ANOVA was performed to identify differential expressed genes using a commercial software package SAS JMP15 Genomics, version 10, from SAS (SAS Institute). A false positive rate of a = 0.05 with FDR correction was taken as the level of significance.

An enrichment score of log_2_FC ∗ *p*-value was calculated and used for functional enrichment in the binary comparisons of either *gnat2* *versus* WT under different light conditions or growth light *versus* light alteration in the different genotypes *via* STRINGdb’s “Proteins with Values/Ranks - Functional Enrichment Analysis” (28).

### Analysis of Metabolites *via* UPLC-FLD

The adenylate status (ATP and ADP) and the NADH/NAD^+^ redox status were analyzed by metabolite fingerprinting using an ultra-performance liquid chromatography (UPLC) system. Approximately 25 mg ground shoot material was extracted with 1 ml 0.1 M HCl by vortexing at 4 °C for 15 min. The resulting homogenates were centrifuged for 10 min at 4 °C and 16,400*g* to remove cell debris. Adenosines were derivatized with chloroacetaldehyde as described in (29) and separated by reversed-phase chromatography on an Acquity HSS T3 column (100 mm × 2.1 mm, 1.7 μm, Waters) connected to an Acquity H-class UPLC system. Prior to separation, the column was heated to 43 °C and equilibrated with five column volumes of buffer A (5.7 mM TBAS, 30.5 mM KH_2_PO_4_ pH 5.8) at a flow rate of 0.6 ml min^−1^. Separation of adenosine derivates was achieved by increasing the concentration of buffer B (2/3 acetonitrile in 1/3 buffer A) in buffer A as follows: 1 min 1% B, 2 min 8% B, 3.2 min 14% B, 9.5 min 50% B, and return to 1% B in 1.5 min. The separated derivates were detected by fluorescence (Acquity FLR detector, Waters, excitation: 280 nm, emission: 410 nm) and quantified using ultrapure standards (Sigma). Data acquisition and processing was performed with the Empower 3 software suite (Waters).

### Quantitative Proteomics and Lysine Acetylome

Frozen leaf tissue was ground in liquid nitrogen to a fine powder and subsequently processed with a modified filter-aided sample preparation protocol using 30 kDa MWCO Amicon filters (Merck Millipore, www.merckmillipore.com) as described in (30). After the tryptic digest, peptides were labeled with tandem mass tags (TMT) according to (31). One channel of a 6plex reagent kit was assigned to a condition (three light conditions (control, high light, darkness) for two genotypes (WT, *gnat2*)). The assignment was randomized within each replicate/kit (Supplemental Data Set 6). The samples were resuspended in TBS buffer (50 mM Tris–HCl, 150 mM NaCl, pH 7.6) and 10 μg peptide of each sample stored for whole proteome analysis. Enrichment for lysine-acetylated peptides was performed as described in (30) using anti-acetyl lysine antibodies bound to agarose beads first from CellSignaling (CS) and subsequently from ImmuneChem (IC). All samples were divided into two equal portions. They were desalted on SDB-RPS stagetips as described in detail in (30). One-half was eluted directly and the other half eluted from the tips as three fractions with 20 μl elution buffer each. Eluted samples were dried in a vacuum concentrator.

### LC-MS/MS Measurement of Proteome and Lysine Acetylome Peptide Samples

Dried peptide samples were resuspended in 2% acetonitrile, 0.1% trifluoroacetic acid and subsequently measured on LC-MS/MS using an EASY-nLC 1200 system coupled to an Exploris 480 mass spectrometer with a FAIMS interface (Thermo Fisher Scientific, www.thermofisher.com). Separation of peptides took place on 20 cm fritless silica emitters with 0.75 μm inner diameter (New Objective, www.newobjective.com) in-house packed with reversed-phase 1.9 μm ReproSil-Pur C_18_-AQ (Dr Maisch GmbH, www.dr-maisch.com). Peptides were separated on 115-min gradients, using 0.1% formic acid as buffer A and 80% acetonitrile, 0.1% formic acid as buffer B. MS^1^ scans were acquired at an Orbitrap Resolution of 120,000 with a scan range (m/z) of 350 to 1200, a maximum injection time of 100 ms, and a normalized automatic gain control target of 300%. For fragmentation, only precursors with charge states 2 to 5 were considered. Up to 20 dependent scans were taken. For dynamic exclusion, the exclusion duration was set to 40 s and a mass tolerance of ± 10 ppm. The isolation window was set to 0.7 m/z with no offset. A normalized collision energy of 36 was used. MS^2^ scans were taken at an Orbitrap resolution of 30,000, with a fixed first mass (m/z) = 100. Maximum injection time was 86 ms and a normalized automatic gain control target of 50%. Turbo TMT was enabled. For full proteome analysis, compensation voltages (CVs) of −45, −60, and −80 were used, while peptides enriched for acK were measured at CVs −40, −55, and −75 in one run. The FAIMS MzXML Generator was used to pre-process raw files with data recorded at several CVs and convert the raw files to mzxml files (32).

### MS Data Analysis of the Proteome and Lysine Acetylome

Interpretation of MS raw data was done using the MaxQuant (MQ) software with the embedded Andromeda search engine (v 2.0.2.0, www.maxquant.org) (33), searching against the latest version of the Araport11 database containing 48,266 entries (v2016/06, www.bar.utoronto.ca/thalemine). Samples (proteome and acetylome) were searched at once, however with dedicated parameter groups with the following settings: A list of common contaminants and a reverse decoy database was enabled for the search. Carbamidomethylation was used as fixed modification. Oxidation of methionine and tryptophan, deamidation of asparagine and glutamine, and N-terminal acetylation were set as variable modification. In addition, lysine acetylation was enabled as variable modification for enriched samples in the respective parameter group. MS^2^-based quantification for TMT 6plex was enabled for all parameter groups. Trypsin was set as protease and maximal two missed cleavages (proteome) or four missed cleavages (acetylome) were accepted. Minimum length of valid peptides was set to seven amino acids. The re-quantify feature was enabled. PSM and protein FDR was 0.01. Minimum scores were set to 0 and 30 for unmodified and modified peptides and delta score to 0 and 6, respectively. A tolerance of peptide of 20/4.5 ppm was allowed for first/main search, while the MS/MS peak tolerance was 20 ppm.

MaxQuant output tables (protein groups and acetyl K sites) were imported to R. The six TMT reporter intensities from all four replicates were log_2_ transformed and each channel was median normalized prior to differential expression analysis *via* Limma. The differences of the log_2_-transformed TMT reporter intensities were tested for 4 *versus* 4 replicates.

The full MaxQuant search results, including all files and folders produced by the software as well as the respective raw data are available *via* ProteomeXchange repositories, providing all necessary information for in depth assessment of the results. Result tables and assigned spectra can be viewed either with MaxQuant’s viewer or PDV (34).

### N-terminal Acetylome

For the N-terminal acetylome analysis, samples were prepared and processed as previously described (35) with few exceptions. Briefly, the proteins extracted from the biological material were labeled on their N-termini and lysine ε-amino groups using N-acetoxy-[2H3] succinimide, before being subjected to trypsin digestion. The peptides obtained were then fractionated on a strong cation exchange chromatography, and the fractions were analyzed, without pooling, on an LTQ-Orbitrap mass spectrometer coupled to a liquid chromatography. The data obtained were processed using Mascot Distiller version 2.6.2 with “Acetyl:2H(3) (K)”, “Carbamidomethyl (C)” as fixed modifications and “Oxidation (M)” as variable modification, “Acetyl (N-term)” and “Acetyl:2H(3) (N-term)” being included in the “Acetylation” quantitation method of Mascot Distiller. Along with these parameters, searching against the Araport-11 protein database, with the parent and fragment mass tolerance respectively defined to 10 ppm and 0.5 Da. The quantitation results, along with each Mascot search output, were then parsed using the EnCOUNTer script to obtain the final N-ter acetylome datasets (36). The complex datasets were manually consolidated to allow for individual result comparison between all the different experimental conditions. Statistical analysis was then performed in the Excel spreadsheets using available built-in formulas and tools. Briefly, all biological replicates of each specific condition were combined, averaging the N-terminal acetylation levels and providing standard deviation across those replicates. After that, NTA levels ratios were calculated and, when possible, *p*-values were determined using a two-tailed *t* test applied to the comparison of two sample groups of similar variances. This allowed to account for both decreases and increases in acetylation levels. Because of the complex experimental design, two distinct types of comparisons were made, either looking for differences related to light treatments in one of the genotypes, or for the effect of the *gnat2* knock-out compared to the WT in one of the three light conditions groups.

## Results

In order to study the plant responses to fluctuations in light intensity with multi-omics approaches (*e.g.* transcriptome, proteome, and acetylome), the *gnat2* and WT plants were grown under the same experimental conditions. Two hours after the onset of light, the one-month-old plants were subjected to three light regimes for 2 h: control light (C), high light (HL), or darkness (D). Immediately after the treatment, rosettes of individual plants were harvested and split for the multi-omics analysis (Fig. 1).

### Energy Status of *gnat2* and WT Plants Upon Short-Light Stress Conditions

First, we focused on the plant energy status by determining ATP, ADP, and AMP levels in WT and *gnat2* and calculating the adenylate energy charge under C as well as under short-term HL and D stress (Supplemental Data Set 1). We found that ATP, ADP, and AMP abundances increased in both WT and *gnat2* after 2 h of treatment with HL and decreased under D independently of the genotype (Supplemental Fig. S1). Nonetheless, the energy charge was not affected by HL and increased slightly upon short-term D in both genotypes (Supplemental Fig. S1). From these findings, we conclude that both genotypes were able to acclimate their metabolism to HL or D comparably.

### Comparison of WT and *gnat2* Transcriptomes

Under control conditions, only 39 transcripts (including *GNAT2*) were differentially regulated between WT and *gnat2* (Supplemental Data Set 2). Of those 39 gene products, eight were downregulated and 31 were upregulated in *gnat2*. Whereas the downregulated transcripts were not enriched in any specific pathways, the upregulated transcripts were mainly involved in translation-related pathways (RNA transport, ribosome biogenesis, aminoacyl-tRNA biosynthesis), suggesting that GNAT2 might be involved in the control of translation.

While the global transcriptomes of the WT and *gnat2* plants differed only weakly under control conditions, pronounced changes in transcript abundances in both genotypes in response to the altered light conditions were detected (Supplemental Data Set 2*A* and Supplemental Fig. S2). However, both genotypes show a similar response to the change in light conditions. This is indicated by high Pearson correlation coefficients, which are 0.90 and 0.89 when comparing the WT *versus gnat2* response to darkness or high light on transcriptome level, respectively (Supplemental Fig. S3, *A* and *B*). Of 27,826 quantified transcripts, only 70 and 71 show a log_2_ FC > 1 or < −1 in response to the respective light change. No functional pathway enrichment could be detected for either altered gene sets using STRINGdb.

### High Light-Dependent Transcriptome Changes in WT and *gnat2*

**Fig. 2:**
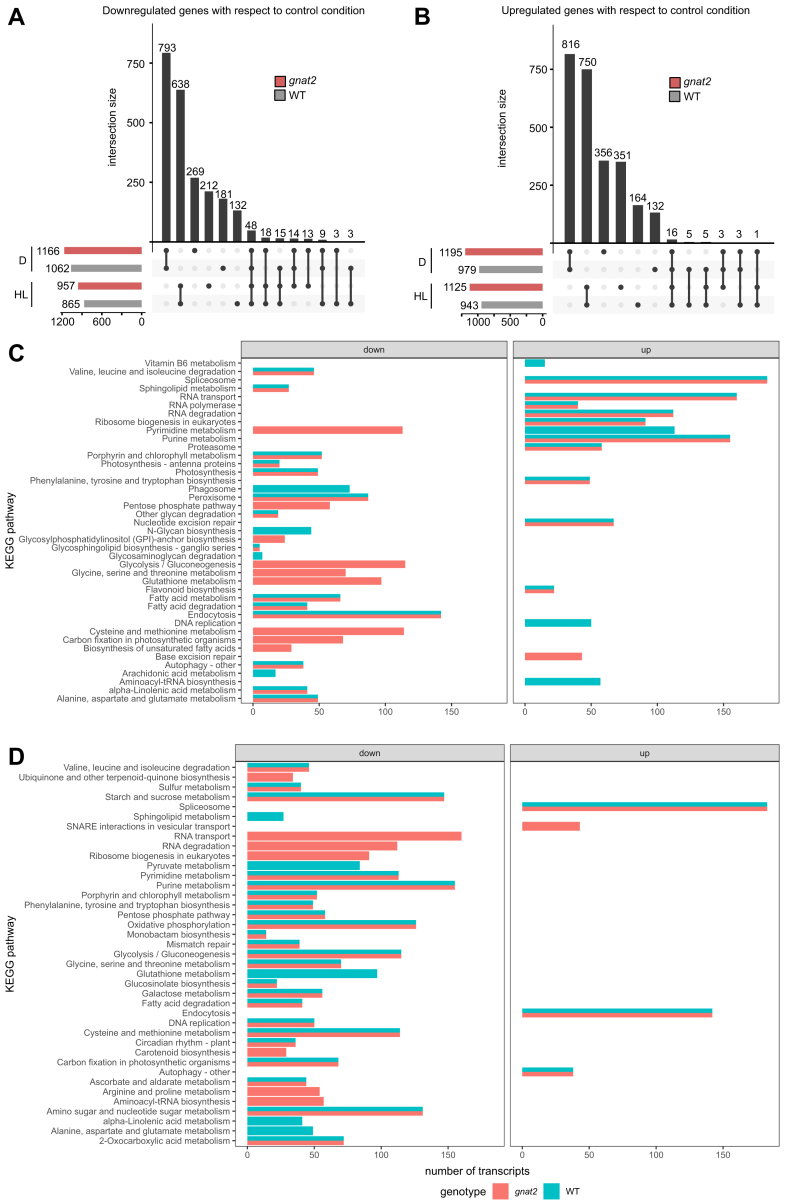
**Transcriptional regulation in WT and *gnat2* upon high light and darkness.** The leaf material from all genotypes (WT and *gnat2*) and treatments (control, high light, and darkness) was subjected to a global transcriptome analysis followed by a functional enrichment analysis with string-db.org. All experiments were performed on four biological replicates. *A* + *B*, UpSet plots, showing the number of genes up- (*B*) or down- (*A*) regulated in WT (*gray* bars) and *gnat2* (*pink* bars) and the respective overlaps upon high light (HL) and darkness (*D*). *C* + *D*, bar charts showing KEGG pathways regulated in WT (*green*) and *gnat2* (*pink*) upon high light (*C*) and darkness (*D*). The size of each bar corresponds to the number of genes annotated to this pathway.

In WT, the expression of 1808 of 27,826 analyzed transcripts was significantly (filter: >2-fold, *p* < 0.05) altered after HL treatment. Of those 1808 transcripts, 943 were upregulated and 865 were downregulated. A similar response to HL was observed in *gnat2*, where 2083 transcripts (1126 up and 957 down) were significantly regulated upon HL stress (Fig. 2, *A* and *B*). When comparing the transcripts regulated in WT and *gnat2* in response to HL, a substantial overlap of 1489 commonly regulated transcripts was identified (82% of regulated transcripts in WT, 71% of regulated transcripts in *gnat2*). The remaining significantly altered transcripts were exclusively regulated in one of the two genotypes (319 in WT and 594 in *gnat2*). There were no antagonistically regulated transcripts between the two genotypes.

The unfiltered data matrix was subjected to an unbiased functional enrichment analysis to identify biologically relevant processes affected by the HL treatment. For this purpose, an enrichment score (ENS = -log_10_(*p*-value) ∗ log_2_(FC)) was calculated for each transcript and the full list of transcripts was analyzed with the functional enrichment tool of STRINGdb (27). The analysis revealed that upon HL treatment, 14 KEGG (Kyoto Encyclopedia of Genes and Genomes) pathways were upregulated (Fig. 2*C* right and Supplemental Data Set 2*B*) and 18 KEGG pathways were downregulated (Fig. 2*C* left and Supplemental Data Set 2*B*) in the WT. Out of these 32 pathways, 24 were also regulated in *gnat2* (Fig. 2*C* and Supplemental Data Set 2*B*), demonstrating that a substantial proportion of the HL response is independent of GNAT2. In both genotypes, the HL treatment decreased transcripts related to photosynthesis, antenna protein biosynthesis, porphyrin/chlorophyll turnover, and lipid metabolism. Processes upregulated in both genotypes were associated with translation, RNA processing, and flavonoid biosynthesis. The specific response of *gnat2* to HL included the downregulation of diverse anabolic reactions like carbon fixation, biosynthesis of unsaturated fatty acids and amino acids, as well as sulfur incorporation (Fig. 2*C* and Supplemental Data Set 2*B*), whereas the base excision repair pathway was the only one specifically upregulated in *gnat2* (Fig. 2*C*, and Supplemental Data Set 2*B*). Our findings indicate that apart from minor changes that specifically occur in *gnat2*, the overall transcriptional response to short-term high-light treatment is very similar between WT and *gnat2*.

### Transcriptome Response to Darkness in WT and *gnat2*

Next, we analyzed the response from control light to darkness. This treatment altered the expression of 2042 transcripts in the WT, of which 979 were upregulated and 1063 were downregulated, respectively (Fig. 2, *A* and *B*, and Supplemental Data Set 2*A*). The *gnat2* mutant displayed a transcriptional response of similar severity (1195 up (Fig. 2*A*) and 1166 down (Fig. 2*B*)). Thousand seven hundred one of these differentially expressed transcripts were commonly regulated in both genotypes (83% of regulated transcripts in WT, 72% of regulated transcripts in *gnat2*). Like in the high-light response, no transcript was antagonistically regulated between both genotypes upon darkness. Again, a minor set of transcripts (341 in the WT and 660 in *gnat2*) was regulated in a genotype-specific manner.

The functional enrichment analysis showed that upon darkness, only transcripts associated with the spliceosome, endocytosis, and autophagy were upregulated in the WT (Fig. 2*D*, and Supplemental Data Set 2*C*). Upregulation of the latter suggests significant nutrient recycling in response to darkness. Loss of GNAT2 did not alter these transcriptional responses and caused an additional upregulation of SNARE-mediated vesicle transport (Fig. 2*D* pink bar, and Supplemental Data Set 2*C*). In both genotypes, most metabolic processes were downregulated upon darkness, including DNA replication, carbon fixation, sulfur incorporation, and porphyrin/chlorophyll biosynthesis (Fig. 2*D* left, and Supplemental Data Set 2*C*). The downregulation of sulfur assimilation can be explained by its dependency on photosynthetic reduction equivalents and its importance for light acclimation (37). The downregulation of DNA replication can be interpreted as a hallmark of decelerated cell division. The dampening of metabolic core pathways is a canonical response of plants to unexpected darkness and is required to maintain the energy charge of leaf cells. Besides these general responses, the *gnat2* mutant also displayed downregulation of transcripts involved in translation-related pathways (RNA transport, ribosome biogenesis, aminoacyl-tRNA biosynthesis) (Fig. 2*D* and Supplemental Data Set 2*C*). These pathways were upregulated in the WT upon illumination with high light; hence our results suggest that GNAT2 might be involved in the light-dependent control of translation.

### High Light-Dependent Proteome Regulations in WT and *gnat2*

The transcriptome results led us to elucidate the effect of light on the proteomes of WT and *gnat2* plants. For this, a TMT-6plex labeling approach followed by quantitative mass spectrometry analyses and MS^2^-based quantification was used and allowed to quantify 5620 protein groups in total (Supplemental Data Set 3).

**Fig. 3:**
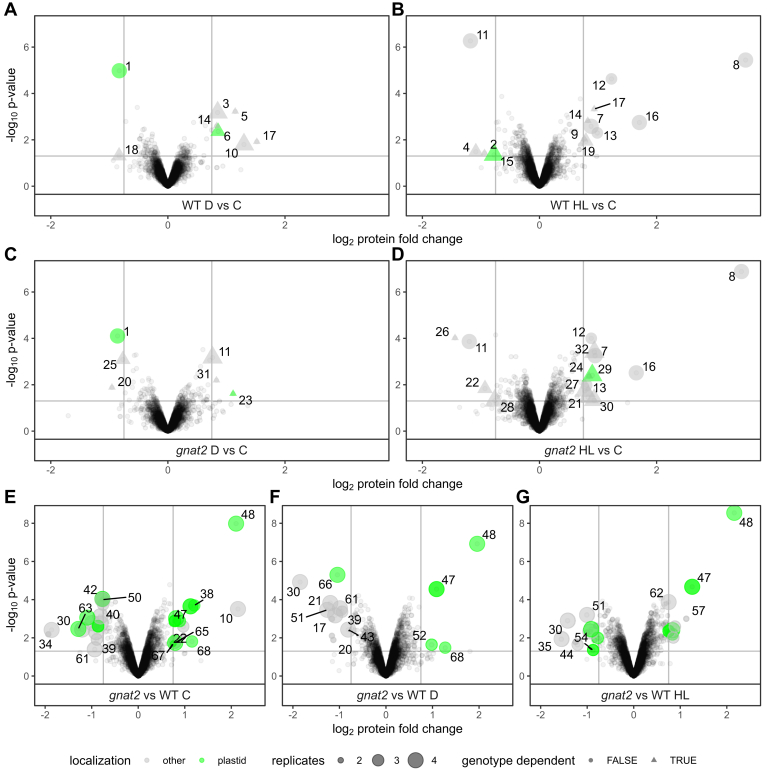
**Quantitative proteome profiling of WT *versus gnat2* under different light conditions.** Protein abundance changes were quantified via TMT labeling and compared for different light conditions (panels *A–D*) or genotypes (panel *E*–*G*). Volcano plots showing expression changes and -log_10_(*p*-values) from LIMMA analysis for protein groups quantified in at least two replicates. log_2_ fold-changes ± 0.75 and *p*-value = 0.05 are indicated as *solid* lines. For significantly altered proteins, either gene names are given (*A–D*) or indicated as numbers (*E*–*G*) and solid symbols are used. *Circles* and *triangles* are scaled in size according to the number of replicates. Plastid-localized proteins are indicated in *green*. Proteins with identical response to the light treatment in either genotype are indicated as *circles* in *A–D*, while proteins with potential genotype-dependent regulation are indicated as *triangles*. Protein accessions in this figure from spot #1 to 68: 1: AT1G61520.3, 2: AT1G64890.1, 3: AT2G41100.7, 4: AT3G07810.1, 5: AT3G10020.1, 6: AT3G10050.1, 7: AT3G12580.1, 8: AT3G46230.1, 9: AT4G15210.2, 10: AT4G17085.1, 11: AT4G31700.1, 12: AT5G11260.1, 13: AT5G12020.1, 14: AT5G16990.1, 15: AT5G43780.1, 16: AT5G48570.1, 17: AT5G56550.1, 18: AT5G56950.1, 19: AT5G61020.2, 20: AT1G01510.1, 21: AT1G13930.3, 22: AT1G14710.2, 23: AT1G32900.1, 24: AT1G74310.1, 25: AT1G75950.1, 26: AT2G41540.4, 27: AT3G07880.1, 28: AT3G47430.1, 29: AT5G01600.1, 30: AT5G15970.1, 31: AT5G20980.2, 32: AT5G56000.1, 33: AT1G01630.1, 34: AT1G02110.1, 35: AT1G27450.4, 36: AT1G30170.1, 37: AT1G32060.1, 38: AT1G43560.1, 39: AT1G71950.1, 40: AT1G76490.1, 41: AT2G06520.1, 42: AT2G14880.1, 43: AT2G24940.1, 44: AT2G45540.6, 45: AT2G46370.3, 46: AT2G47400.1, 47: AT3G18890.1, 48: AT3G21055.1, 49: AT3G21055.2, 50: AT3G26450.1, 51: AT3G29075.1, 52: AT3G52950.2, 53: AT3G56240.1, 54: AT3G63140.1, 55: AT4G21445.1, 56: AT4G23670.1, 57: AT4G30810.1, 58: AT4G32050.1, 59: AT4G33360.2, 60: AT4G36210.3, 61: AT4G37300.1, 62: AT4G37930.1, 63: AT5G13270.1, 64: AT5G19240.1, 65: AT5G28500.1, 66: AT5G66090.1, 67: ATCG00630.1, 68: ATCG01100.1. TMT, tandem mass tags.

Upon HL treatment, both WT (Fig. 3*A*) and *gnat2* mutants (Fig. 3*C*) showed a strong accumulation of heat shock response proteins, such as the heat shock protein (HSP) 20 family proteins HSP17.4 (#8 in Fig. 3) and HSP17.6II (#13), HSP70 (#7), HSP81.4 (#32), HSP101 (#24), and the co-chaperone ROF2 (#16), which is a carboxylate clamp-tetratricopeptide protein, predicted to interact with HSP70 and HSP90 proteins (38). Several of these proteins are involved in the cytosolic response to heat as well as high light stress to protect translation factors, prevent protein aggregation, and enable proteasomal degradation of damaged proteins (39, 40, 41). Both genotypes also showed a twofold upregulation in HY5 (#12 in Fig. 3) protein level. HY5 is a basic leucine zipper transcription factor, which is critical for protection from high light–associated DNA damage by anthocyanin accumulation (42). Among the downregulated proteins upon HL treatment, the ribosomal protein S6A (RPS6A, #11) was identified in both genotypes. RPS6A is important for light- and photosynthesis-enhanced translation by its association with polysomes in its phosphorylated state and connected to the TOR pathway (43). Interestingly, two proteins involved in oxidative stress tolerance, the chromatin-associated factor OXS3 (#17) and a 2-alkenal reductase (#10, AT4G17085.1) involved in NADPH-dependent detoxification of reactive carbonyls (44, 45), were found more than two-fold upregulated in the WT upon HL stress (Fig. 3*A*), but not in the *gnat2* mutant (Fig. 3*C*). In contrast, a hydroxyproline-rich glycoprotein family protein of unknown function (#22, AT1G14710.2) was systematically downregulated by more than 2-fold in *gnat2* but not in WT upon HL treatment.

### Darkness-dependent Proteome Regulations

Upon 2 h of darkness, six proteins were significantly and exclusively upregulated in their abundance in WT (Fig. 3, *B* and *C*). Among them, we identified TOUCH 3 (#3 in Fig. 3), a calmodulin-like 4 protein already known to be transcriptionally induced by darkness (46), and the two oxidative stress-related proteins OXS3 (#17) and 2-alkenal reductase (#10). The nucleosome assembly protein 1:3 (NFA3, #18), which is vital for the protection of DNA damage, was only downregulated in WT plants. The plastid methionine synthase 3 protein (#31), the ribosomal protein S6A (RPS6A, #11), and the granule-bound starch synthase 1 (GBSS1, #23) were exclusively upregulated in the *gnat2* mutant upon darkness, while the S phase kinase-associated protein 1 (UIP1, #25) was significantly downregulated in its abundance. Only one protein, the chlorophyll binding protein LHCA3 (#1) of photosystem I, already known to be involved in the response to light stimuli (47), was decreased in abundance in both genotypes.

### Differences in Protein Abundances Between WT and *gnat2*

Changes in protein abundances between WT and *gnat2* were mostly independent of the stress treatment (Fig. 3, *E*–*G*). The most striking differences were detected in more than 16-fold upregulation of the nuclear-encoded photosystem II 5 kDa protein (PsbTn, #48 in Fig. 3) in *gnat2*, which was found to be constitutively upregulated in its abundance. PsbTn was recently shown to be critical for light acclimation (48). In addition, the protein TRANSLOCON AT THE INNER ENVELOPE MEMBRANE OF CHLOROPLASTS 62 (TIC62, #47), which is fundamental for the anchoring of the chloroplast ferredoxin-NADPH reductase to the thylakoid membrane (49), was more than 2-fold upregulated in *gnat2* compared to WT under all growth conditions. Generally, the transcriptome changes were more pronounced than those related to the proteome. Due to the short 2 h light treatments, the transcriptome changes were not yet propagated to the proteome except for a few gene products (Supplemental Fig. S4 and Supplemental Data Set 3).

### The N-terminal Acetylome under Short-Term Changes in Light Conditions

Recently, it has been suggested that plastid NTA might be related to oxidative damage because of the high levels of NTAed subunits of many plastid complexes correlated with oxygenic photosynthesis (25). Moreover, although NTA is a widespread modification in plastids, it was proposed that plastid NTA yield might depend on environmental stimuli, for example, light (1). Nonetheless, no information was available on the processing of the N-termini of plastid proteins under different light conditions so far. Therefore, to get an insight into the possible dynamics of the N-terminal acetylome in Arabidopsis and to decipher the influence of NTA in response to different light conditions, we performed a global NTA quantitative analysis on the very same sample set as above (Fig. 1). With the SILProNAQ procedure (35, 50), we identified a total of 1783 nonredundant proteoforms in WT plants (Supplemental Data Set 4 and Supplemental Table S1). Amid all identified proteoforms, the acetylation yields were quantified for 761 of them.

**Fig. 4:**
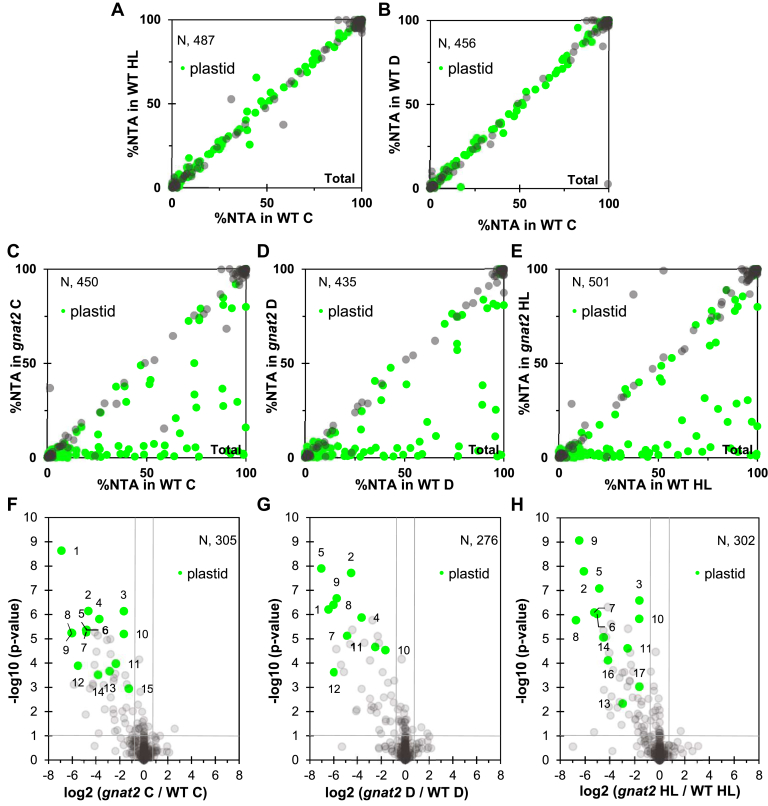
**Growth condition–dependent NTA analysis of *gnat2* plantlets compared to WT.** Correlation graphs representing the N-terminal acetylation yields of proteins that have been quantified in both the WT control and WT high light conditions (*A*) or WT control and WT dark (*B*), which amount to 487 and 456 N-termini, respectively. These plots show that the NTA levels are similar in WT samples, regardless of the light conditions used during the treatment, which means the samples could be combined for further analysis. Panel *C–E*: Correlation graphs representing the N-terminal acetylation yields of proteins that have been quantified in both the WT and *gnat2* samples in either control (*C*), dark (*D*), or high light (*E*) conditions, amounting to 450, 435, and 501 N-termini, respectively. All the data points on the lower-right part of the graph represent the proteins with decreased NTA yields in the *gnat2* samples compared to the corresponding WT. Proteins located in the plastid, regardless of NTA levels, are colored in *light green*. *F*–*H*, volcano plots representing the NTA variations between *gnat2* samples and WT, for each of the three growth conditions. The *p*-value threshold was set at 0.1, and the log_2_ fold-change to ± 0.75. The N-termini that satisfy both of these conditions and have a decreased NTA level of at least 40% in *gnat2* are numbered (see Supplemental Tables S2–S4, respectively). Proteins located in the plastid and displaying a significant decrease of NTA level are colored in *light green*. Protein accessions (and start positions) for (*F*–*H*) of this figure from spot #1 to 17: 1: AT1G64770.1 (19), 2: AT4G27440.1 (68), 3: AT4G24830.1 (75), 4: AT3G57560.1 (51), 5: AT2G24820.1 (51), 6: AT1G03630.1 (69), 7: AT1G16080.1 (45), 8: AT3G54050.1 (60), 9: AT3G03630.1 (57), 10: AT3G54050.1 (61), 11: AT4G17300.1 (60), 12: AT4G35250.1 (65), 13: AT3G63410.1 (51), 14: AT1G45474.1 (33), 15: AT2G29630.1 (55), 16: AT5G45390.1 (66), 17: AT4G30950.1 (70).

Of the 761 N-termini quantified in the leaves of WT under control conditions, only 112 started with the initiator methionine (iMet, 15%), 280 (37%) were subject of iMet removal, and 369 were generated by a cleavage of the transit peptide. Sixty two percent of N-termini undergoing removal of iMet were found fully NTAed (NTA yield >95%), whereas 14% were partially NTAed (NTA yield between 5 and 95%). Concerning the N-termini retaining the iMet, 79% were fully NTAed and only 12% partially NTAed. As previously observed, among the N-termini with processing downstream of position 2, only 8% were fully NTAed and 37% partially NTAed. When these parameters were analyzed in the samples arising from plants treated with D or HL, no significant difference was observed with the plants from control conditions, and all three conditions of each group followed the same pattern of NTA yield variations, regardless of the NTA position (Fig. 4, *A* and *B*, and Supplemental Data Set 4).

**Fig. 5:**
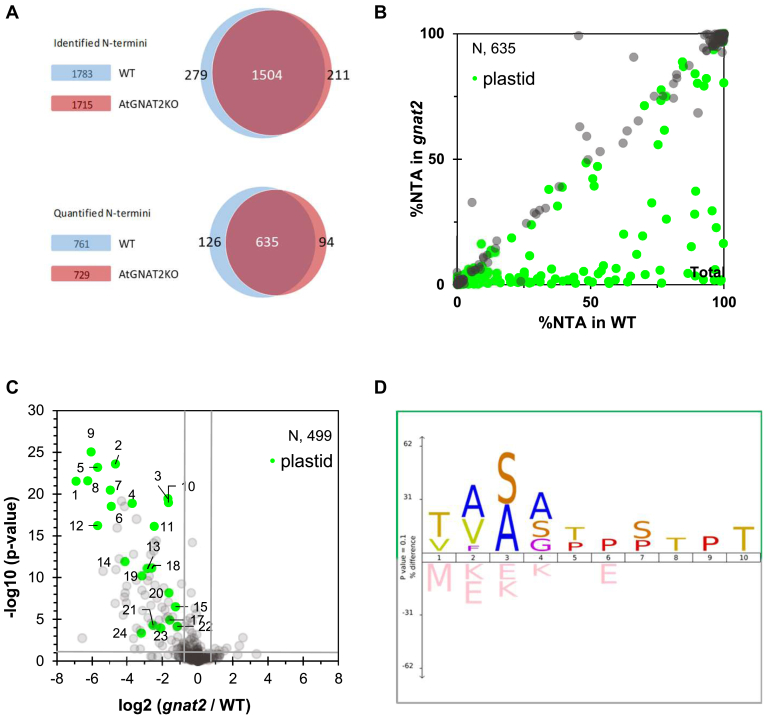
**Impact of *gnat2* on *in vivo* NTA substrates.***A*, Venn diagrams representing the N-termini either identified (*upper*) or quantified (*lower*) in WT and *gnat2* samples. In each case, more than 80% of N-termini were shared between the two conditions, allowing for a reliable comparison. *B*, correlation graphs representing the N-terminal acetylation yields of proteins that have been quantified in both WT and *gnat2* samples independently of the growth condition, amounting to 635 N-termini. All the data points on the lower-right part of the graph represent the proteins with decreased NTA yields in the *gnat2* samples. Proteins located in the plastid, regardless of NTA levels, are colored in *light green*. *C*, volcano plot representing the NTA variations between *gnat2* and WT grouped samples. The *p*-value threshold was set at 0.1 and the log_2_ fold-change to ± 0.75. The N-termini that satisfy both of these conditions and have a decreased NTA level of at least 40% in *gnat2* are numbered (see below). Proteins located in the plastid and displaying a significant decrease of NTA level are colored in *light green*. *D*, iceLogo representation comparing the N-ter sequences with a 40% decrease minimum of NTA yield to those with less than a 5% variation, when comparing the *gnat2* mutants to the WT samples, after combining all the entry values from the corresponding light condition groups (see below and Table 1). Protein accessions (and start positions) for (*C*) of this figure from spot #1 to 24: 1: AT1G64770.1 (19), 2: AT4G27440.1 (68), 3: AT4G24830.1 (75), 4: AT3G57560.1 (51), 5: AT2G24820.1 (51), 6: AT1G03630.1 (69), 7: AT1G16080.1 (45), 8: AT3G54050.1 (60), 9: AT3G03630.1 (57), 10: AT3G54050.1 (61), 11: AT4G17300.1 (60), 12: AT4G35250.1 (65), 13: AT3G63410.1 (51), 14: AT1G45474.1 (33), 15: AT2G29630.1 (55), 17: AT4G30950.1 (70), 18: AT3G02780.1 (46), 19: AT1G16080.1 (44), 20: AT1G18170.1 (29), 21: AT5G13630.1 (50), 22: AT1G09795.1 (57), 23: AT4G13430.1 (48), 24: AT3G13490.1 (48).

Since *gnat2* was recently shown to strongly affect plastid NTA in plants grown in standard conditions (2), we asked whether this phenotype was intensified or reduced when the plants were treated with D or HL. Hence, we performed a global NTA quantitative analysis of *gnat2* grown in the same light conditions (C, D, and HL) and compared their retrieved N-termini and NTA yield to WT (Fig. 4, *C*–*H* and Supplemental Data Set 4). Comparison of *gnat2* with WT plants under C confirmed the decrease of plastid protein NTA when GNAT2 is inactivated (Fig. 4*C*, Supplemental Data Set 4, and Supplemental Table S2). More than 20 N-termini with significant decreases of acetylation (>40%), including about 15 with statistical significance (|log_2_(ratio)| > 1, *p*-value < 0.1), were retrieved in *gnat2* under standard growth light conditions (Fig. 4*D* and Supplemental Table S2). Identical trends were observed when comparing WT and *gnat2* under D and HL conditions (Fig. 4, *D*, *E*, *G*, and *H* and Supplemental Tables S3 and S4). Indeed, many N-termini were quantified in at least two groups with similar NTA variations (Supplemental Tables S2–S4) and when comparing each light condition of WT and *gnat2*, no significant NTA variations could be observed. These results indicate that the N-terminal acetylome is not influenced by short-term light changes and that the different light conditions in terms of NTA are as similar as biological replicates. Treating the different light conditions as biological replicates increased the number of identified and quantified N-termini (*e.g.* yielded 87% overlap between WT and *gnat2*, Fig. 5*A*). This allowed us to get a more profound analysis of the impact of *gnat2* KO background as we could validate further proteins (Fig. 5, *B*, and *C* and Supplemental Data Set 4). Intriguingly, proteins in which NTA was strongly reduced in the *gnat2* background were involved in photosynthesis, amino acid biosynthesis, plastid translation, and RNA interactions (Table 1). The iceLogo representation of the N-terminal sequences with a 40% decreased NTA yield (with less than a 5% variation) in *gnat2* compared to WT shows that proteins starting with Thr or Val are the most favored substrates of GNAT2 (Fig. 5*D*). NatB-like substrates (MetAsp/Glu) are clearly insensitive to the loss of GNAT2. In addition, residues at positions 1 and 3 seem to play an important role for GNAT2 substrate recognition, with Ala and Ser largely preferred, as well as Val at position 1 (Fig. 5*D*). Finally, alterations in NTA level did not lead to significant changes in total protein abundance during the short-term light treatments, as well as in the *gnat2* mutant when compared to the WT (Supplemental Fig. S5).

**Table 1:** Proteins displaying significant NTA decrease (at least 40%) in *gnat2* compared to WT samples, after combining all the entry values from all the corresponding light condition into two global WT and *gnat2* groups; entries with a plot number were quantified in both replicates of each condition, allowing the calculation of a *p*-value

Plot #	Araport-11 accession	Entry name	Subcellular localization	Acetylation position	N-1 residue	N-terminal sequence	%NTA (WT)	%NTA (gnat2)	% NTA Diff.(gnat2-WT)	Protein expression Stability	KAcRatio (gnat2/WT)
9	AT3G03630.1	CYSK4_ARATH	plastid	57	G	AISGKSSTGT	98.7 ± 1.5	1.5 ± 0.6	−97.2	+	n.i.
2	AT4G27440.1	PORB_ARATH	plastid	68	Q	TAATSSPTVT	89.3 ± 2.2	3.5 ± 0.6	−85.8	+	n.i.
5	AT2G24820.1	TIC55_ARATH	plastid	51	S	AVAGTAVSDQ	96.4 ± 1.0	1.9 ± 2.4	−94.6	+	n.i.
8	AT3G54050.1	F16P1_ARATH	plastid	60; 323 (K)	M	AVAADAAETK	44.3 ± 1.5	0.6 ± 0.2	−43.8	+	0.975
1	AT1G64770.1	PNSB2_ARATH	plastid	19	S	SISAPQTQT	76.6 ± 1.4	0.6 ± 0.3	−76.0	+	n.i.
7	AT1G16080.1	C0Z2K9_ARATH	plastid	45; 275 (K)	A	AASAATAKKL	59.2 ± 2.2	1.9 ± 0.7	−57.4	+	1.308
3	AT4G24830.1	ASSY_ARATH	plastid	75	A	VLSGDGTALT	95.6 ± 0.9	29.5 ± 2.0	−66.1	+	n.i.
10	AT3G54050.1	F16P1_ARATH	plastid	61; 323 (K)	A	VAADAAETKT	89.1 ± 2.6	28.1 ± 1.7	−60.9	+	0.975
4	AT3G57560.1	NAGK_ARATH	plastid	51; 91 (K)	A	TVSTPPSIAT	76.0 ± 1.4	5.8 ± 0.8	−70.2	+	0.836
6	AT1G03630.1	PORC_ARATH	plastid	69; 334 (K)	Q	TVTATPPANE	49.2 ± 1.5	1.6 ± 0.2	−47.6	+	1.081
12	AT4G35250.1	HC244_ARATH	plastid	65	C	SAAAVNLAPG	98.9 ± 1.0	1.9 ± 0.8	−97.1	+	n.i.
11	AT4G17300.1	SYNO_ARATH	plastid	60	C	TAVSESLGSG	67.3 ± 2.8	12.1 ± 1.4	−55.2	+	n.i.
14	AT1G45474.1	LHCA5_ARATH	plastid	33	K	AAGGGINPTV	70.5 ± 2.7	4.1 ± 1.4	−66.4	+	n.i.
18	AT3G02780.1	IDI2_ARATH	plastid	46; 144 (K)	R	AFSGTAMTDT	99.8 ± 0.3	16.4 ± 03	−83.4	+	0.862
13	AT3G63410.1	BQMT_ARATH	plastid	51	C	SSSSVSSSRP	54.8 ± 2.6	7.6 ± 1.7	−47.2	+	n.i.
19	AT1G16080.1	C0Z2K9_ARATH	plastid	44; 275 (K)	M	AAASAATAKK	47.4 ± 1.2	5.3 ± 0.5	−42.1	+	1.308
20	AT1G18170.1	FK172_ARATH	plastid	29	Y	ASSSNPPEPE	62.4 ± 2.1	20.2 ± 1.4	−42.2	+	n.i.
15	AT2G29630.1	THIC_ARATH	plastid	55	A	TLTFDPPTTN	89.4 ± 1.7	37.3 ± 1.4	−52.1	+	n.i.
17	AT4G30950.1	FAD6C_ARATH	plastid	70	A	VAAPVAPPSA	78.4 ± 2.6	26.1 ± 1.1	−52.3	+	n.i.
21	AT5G13630.1	CHLH_ARATH	plastid	50	S	AVSGNGLFTQ	87.8 ± 5.7	15.2 ± 6.7	−72.6	+	n.i.
22	AT1G09795.1	HIS1B_ARATH	plastid	57	C	VSNAQKSVLN	72.9 ± 1.2	32.6 ± 0.9	−40.3	+	n.i.
23	AT4G13430.1	LEUC_ARATH	plastid	48	S	VMAPQKDRSP	97.1 ± 0.1	22.8 ± 3.7	−74.3	+	n.i.
24	AT3G13490.1	SYKM_ARATH	plastid, mitoch	48	S	AASSSSSSAT	59.6 ± 3.4	6.5 ± 0.0	−53.0	+	n.i.
	AT1G74970.1	RR9_ARATH	plastid	53	A	TVSAPPEEEE	96.7	6.1	−90.6	+	n.i.
	AT1G68590.1	RRP31_ARATH	plastid	50; 134 (K)	L	AAPETLTAET	92.3	2.0 ± 0.1	−90.3	+	0.949
	AT4G33470.1	HDA14_ARATH	plastid	45	C	SFSTEKNPL	86.5	4.5 ± 1.1	−82.0	+	n.i.
	AT5G22800.1	SYAP_ARATH	plastid	56	A	KSSSVSVQPV	63.9	1.1 ± 0.2	−62.8	+	n.i.
	AT2G25840.1	SYWM_ARATH	plastid	53	C	SVATDDTSPS	64.9 ± 2.0	3.4	−61.6	+	n.i.
	AT3G54050.1	F16P1_ARATH	plastid	63; 323 (K)	A	ADAAETKTAA	57.0	0.5 ± 0.2	−56.5	+	0.975
	AT3G13490.1	SYKM_ARATH	Plastid, mitoch	46	C	ASAASSSSSS	56.7	1.3 ± 0.3	−55.4	+	n.i.
	AT1G59840.1	CCB4_ARATH	plastid	34	A	SSSSTSQKPK	69.5	19.4	−50.1	+	n.i.
	AT2G26670.1	HMOX1_ARATH	plastid	57	A	TTAAEKQKKR	50.6	1.1 ± 0.2	−49.5	+	n.i.
	AT1G484A600A.1	78W3G7_ARATH	plastid	62	C	SSSQSDSRPE	52.9	4.4 ± 1.5	−48.5	+	n.i.
	AT1G31180.1	LEU33_ARATH	plastid	35	C	AAASPVKKRY	53.9	5.5 ± 1.4	−48.5	+	n.i.
	AT5G16440.1	IDI1_ARATH	plastid	56	S	AVTMTDSNDA	43.7	3.0 ± 1.5	−40.7	+	n.i.

Other entries were only quantified in a single replicate of at least one condition. Unless specified otherwise, all acetylated positions are protein N-termini. The plot number (Plot #) is referring to the labels used in Figures 4 and 5.

n.i. entries not identified in the experiment.

### Lysine Acetylome Analysis under the Different Light Conditions

**Fig. 6:**
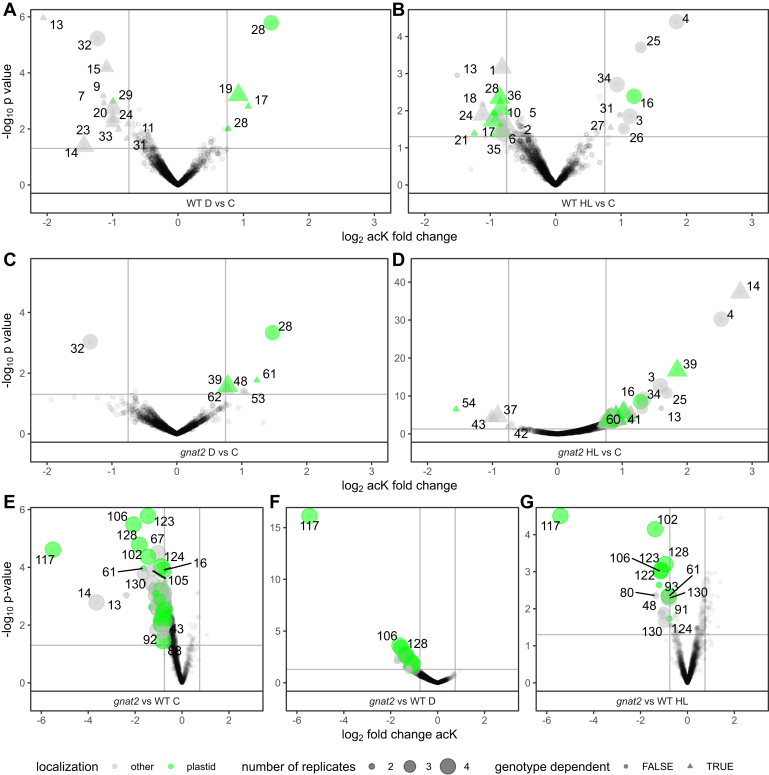
**Quantitative lysine acetylation (acK) profiling.** acK abundance changes were quantified *via* TMT labeling and compared either for different light conditions (panels *A–D*) or genotypes (panel *E–G*). Volcano plots showing abundance changes and -log_10_(*p*-values) from LIMMA analysis for acK sites quantified in at least two replicates. log_2_ fold-changes ± 0.75 and *p*-value = 0.05 are indicated as *solid lines*. For significantly altered acK sites, the corresponding gene names are given (*A–D*) or indicated as numbers; *solid symbols* are used and both *circles* and *triangles* are scaled in size according to the number of replicates. Plastid-localized proteins are indicated in *green*. Sites with identical response to the light treatment in either genotype are indicated as *circles* in *A–D*. Protein accessions in this figure from spot #1 to 136: 1: AT1G66200.1, 2: AT1G70310.1, 3: AT1G74310.1, 4: AT1G06040.1, 5: AT1G77800.7, 6: AT1G79550.2, 7: AT2G20420.1, 8: AT2G27020.1, 9: AT2G28720.1, 10: AT2G30620.1, 11: AT2G30950.4, 12: AT3G10520.1, 13: AT3G14540.2, 14: AT3G16785.6, 15: AT3G22430.1, 16: AT3G47070.1, 17: AT1G12900.1, 18: AT3G55280.3, 19: AT4G25050.1, 20: AT4G29790.1, 21: AT4G32520.2, 22: AT4G34870.1, 23: AT5G02960.1, 24: AT5G07090.3, 25: AT5G11260.1, 26: AT5G15850.1, 27: AT1G20670.1, 28: AT1G54580.1, 29: AT3G55800.1, 30: AT3G62030.1, 31: AT5G14370.1, 32: AT1G26630.1, 33: AT1G32750.1, 34: AT1G44575.3, 35: AT1G52740.1, 36: AT1G56190.1, 37: AT1G65660.1, 38: AT1G79600.1, 39: AT2G05100.1, 40: AT5G60390.3, 41: AT2G39730.2, 42: AT2G45290.2, 43: AT3G04920.2, 44: AT3G09440.4, 45: AT3G15970.2, 46: AT3G17930.1, 47: AT3G44110.1, 48: AT3G62120.3, 49: AT4G01150.1, 50: AT4G20850.1, 51: AT4G38100.2, 52: AT5G01530.1, 53: AT5G07350.1, 54: AT5G13510.1, 55: AT5G15200.1, 56: AT5G26000.1, 57: AT5G27670.1, 58: AT5G47840.2, 59: AT5G59910.1, 60: ATCG00490.1, 61: AT1G51400.1, 62: AT1G75750.1, 63: AT3G46780.1, 64: AT5G13630.1, 65: AT5G20720.4, 66: AT5G66570.1, 67: AT1G29470.2, 68: AT1G30480.1, 69: AT1G50620.1, 70: AT1G51060.1, 71: AT1G55490.5, 72: AT1G61010.5, 73: AT1G61730.1, 74: AT1G65930.1, 75: AT1G68790.1, 76: AT1G72150.1, 77: AT2G18740.2, 78: AT1G07320.4, 79: AT4G38970.1, 80: AT5G59970.2, 81: AT2G31670.1, 82: AT3G18780.3, 83: AT1G01090.1, 84: AT2G46820.2, 85: AT2G47610.1, 86: AT1G09340.1, 87: AT3G02350.1, 88: AT3G08940.2, 89: AT5G02500.1, 90: AT3G12390.1, 91: AT3G13930.1, 92: AT3G15790.3, 93: AT3G21055.1, 94: AT3G25860.1, 95: AT3G26070.1, 96: AT3G26450.1, 97: AT3G44310.3, 98: AT3G48420.1, 99: AT3G48930.1, 100: AT3G52250.2, 101: AT3G54560.2, 102: AT3G56010.1, 103: AT3G04120.1, 104: AT3G63140.1, 105: AT4G05180.2, 106: AT4G13500.1, 107: AT4G23850.1, 108: AT4G24280.1, 109: AT4G34240.4, 110: AT5G09510.1, 111: AT5G10010.1, 112: AT5G22450.3, 113: AT5G22800.1, 114: AT5G38420.1, 115: AT5G42980.1, 116: ATCG00130.1, 117: ATCG00270.1, 118: ATCG00350.1, 119: ATCG00680.1, 120: ATCG00800.1, 121: AT1G20020.1, 122: AT1G20340.1, 123: AT1G52510.1, 124: AT1G54630.1, 125: AT1G55670.1, 126: AT1G71500.1, 127: AT1G74970.1, 128: AT2G05310.1, 129: AT3G15190.1, 130: AT3G21055.2, 131: AT4G01690.1, 132: AT4G21280.1, 133: AT5G35630.3, 134: AT1G03630.2, 135: AT1G42970.1, 136: AT3G12780.1. TMT, tandem mass tags.

Previous studies have shown that photosynthetic proteins are overrepresented among the cellular acK proteins next to histones (51). In addition, it was reported that lysine acetylation affects the activities of several Calvin-Benson cycle enzymes, such as RuBisCO and RuBisCO activase (6, 7, 51). While an increase in RuBisCO lysine acetylation on K334 was reported to occur in the darkness within the diurnal cycle of Arabidopsis plants (7), the dynamic response of lysine acetylation to unexpected changes in light conditions has not been investigated so far. Hence, we analyzed the light-dependent response of the lysine acetylome in response to D and HL, as well as the specific role of GNAT2 in this response. For the lysine acetylome analysis, TMT-labeled peptides from each growth condition were pooled and enriched for peptides carrying acetylated lysines (52). The samples were analyzed by mass spectrometry and acK changes were quantified in both genotypes under the different light conditions. In total, we detected 2560 acK sites (Supplemental Data Set 5). Figure 6 summarizes the overall response in altered lysine acetylation of the plants. In WT plants, 26 and 17 acK sites showed a significant change in their abundances (*p*-value <0.05, fold-change >0.75 or < −0.75, n > 2–4) upon 2 h HL or D treatment, respectively (Fig. 6, *A* and *B*). Of those, eight and seven acK sites were derived from plastid proteins (indicated in green). In comparison, the *gnat2* plants showed a more pronounced deregulation in the lysine acetylome after 2 h HL treatment with 50 acK sites upregulated, while only nine acK sites were also significantly upregulated in the WT. From the 54 significantly regulated acK sites in total, 16 were derived from plastid proteins (Fig. 6*D*, highlighted in green). For example, the large subunit of RuBisCO (#60 in Fig. 6) and RuBisCO activase (#41) were increased in lysine acetylation, while their modification status was not significantly altered in WT plants under the different light conditions. However, proteins from other subcellular localizations, such as phospholipase D Z1 (#14) which resides at the plasma membrane, were also significantly more acetylated in *gnat2* under HL conditions, while the site was downregulated in WT. Only seven acK sites were significantly changed in their abundances after 2 h D in *gnat2* plants (Fig. 6*C*), among which two were overlapping with WT plants from the same treatment. In *gnat2*, the light harvesting protein LHCB2.1 (#39) was strongly increased in its acK status under both D and HL compared to C conditions, while in WT, much milder (though significant) increases in acK fold-changes were observed. This is interesting in the context that *gnat2* mutants fail to perform state transitions (13). In both genotypes, several proteins involved in transcriptional regulation, such as HY5 (#25 in Fig. 6), CONSTANS-like 1 (#26), SALT TOLERANCE (STO, #4), histone H2A (#70), and the CLEAVAGE AND POLYADENYLATION SPECIFICITY FACTOR 73-I (#72) were found more acetylated under 2 h HL conditions compared to C (Fig. 6, *B*, *D*, and *G*). When transferred to 2 h D, plants of both genotypes respond with a strong acetylation of K55 of acyl carrier protein 2 (ACP2, #28), localized to chloroplasts (Fig. 6, *A*, *C*, and *F*). While the respective site is conserved in ACP4 (#19), no modification is detected on K52. The protein sequences of ACP2 and 4 are 48% identical, however not aligned in the first 48 AA representing their respective transit peptides. The acetylation of the conserved K67 in ACP4 (#19) is more pronounced in WT plants (Fig. 6*A*, *p* < 0.05), which might be explained due to the varying sequence context in ACP2 (#28) and 4 for this particular residue. Both these ACPs are known to be expressed in leaves. Generally, the lysine acetylome was responsive to the light treatment as shown in the deregulation of several acetylation sites on proteins from photosynthesis and other cell functions. While there was little change in protein abundance levels, the lysine acetylation was primarily responsive to the different light treatments (Supplemental Fig. S6, *A* and *B*).

A direct comparison of the effect of *gnat2* relative to WT shows a more than 30-fold downregulation in acK level of the photosystem II PSBD protein (#117, Fig. 6, *E*–*G*), which is dependent on the genotype but independent of the light conditions. This essential photosynthetic protein, forming the core of PSII together with the D1 protein, is not altered in its protein abundance when comparing *gnat2* to WT. This contrasts with PSBTN, which is strongly upregulated in its abundance in *gnat2*, while its acK status is slightly decreased in *gnat2* relative to WT (Supplemental Fig. S6). While the acK PSBD protein has not been identified in our previous study, several other proteins were confirmed as GNAT2 substrate proteins in this lysine acetylome analysis, such as PSBH, KEA1/2, and two small unknown thylakoid membrane proteins (Supplemental Fig. S7). When correlating changes in acK and NTA status of the different light treatments in WT and *gnat2* mutants, also here it was apparent that the lysine acetylome is responsive to light while nearly no variation in NTA levels were observed (Supplemental Fig. S8, *A*–*D*). In response to *gnat2* compared to WT, there is only the PSBD protein which is simultaneously downregulated in its acK and NTA level (acK down-regulated by 45-fold, NTA down-regulated by 20%) independent of the light treatment (Supplemental Fig. S8, *E*–*G*).

## Discussion

The rapid development of multi-omics approaches has made it possible to acquire and analyze multidimensional big data (*e.g.* representing protein and nucleic acid modifications, proteomes, transcriptomes, and metabolomes) of entire organisms. However, several challenges persist before we can fully take advantage of these approaches. When the data are collected from various sources, it can be challenging to connect the information with each other with the aim to provide an integrative overview about the cellular or organism wide responses. Especially co- and post-translational protein modifications provide essential mechanisms for regulating protein activities and functions. However, the systematic evaluation of their relevance is at its infancy, and even less is known about the interplay of different co-occurring protein modifications and how they respond to different stimuli (53). There are only a few studies where the dynamics of different protein modifications are coupled with other omics data in response to specific signals to systemically address cell signaling processes and physiological responses to the environment (41, 54, 55, 56).

In this study, we provide the first comprehensive overview of how the transcriptome, proteome, lysine, and N-terminal acetylome are simultaneously orchestrated in response to short-term D and HL treatments in Arabidopsis WT and a *gnat2* KO mutant line. Specifically, we investigate how the two different types of acetylations catalyzed by one enzyme—here GNAT2—in the chloroplast distinctively respond to short-term changes in light conditions.

Light alterations on the long run, including seasonal variations or night-day transitions, are one of the major regulatory factors controlling germination, growth, and general fitness of plants. In addition, within a single day, hour after hour, plants need to constantly acclimate to short- and longer-term changes in light intensity. Plants avoid photo-oxidative damage by multiple strategies, including changes in the absorption cross-section of the light-harvesting antenna associated with either PSII or PSI (*i.e.*, state transitions (57)), induction of thermal dissipation of absorbed excess energy (*i.e.*, non-photochemical quenching) (58, 59), activation of the malate valve for the export of excess reducing equivalents (60), dissipation of excess energy in mitochondria (61), and induction of the ROS scavenging network (62). Intense illumination also results in alkalization and Mg^2+^ accumulation in the chloroplast stroma, which activates the Calvin-Benson cycle. All these actions allow transient reconfiguration of primary and secondary metabolism of the plant through retrograde signaling cascades, which coordinate chloroplast function and nuclear gene expression (63). Many studies are available on the acclimation to long-term light changes, but less is known about how the proteome and different PTMs respond in a coordinated manner to short term changes in light availability. A recent study investigated the effect of short-term HL treatment from 2 min to 72 h on both the transcriptome and metabolome levels and on the protein redoxome in Arabidopsis (41). While strong regulations were observed on the metabolome level, which must be directed by changes in protein activities, no major changes were observed on the dithiol-based regulation of the proteome (41).

In our study, we observed the upregulation of adenosine metabolites after the 2 h exposure to HL. The opposite tendency was revealed under the 2 h exposure to D. This is in agreement with previous data showing that metabolism of photosynthesizing organisms changes immediately upon alteration of photosynthetic conditions such as light (41, 54). However, the preservation of the energy status upon HL illumination suggests that our short-term light treatment did not lead to substantial photo-inhibition, which is in line with a recent study showing only a slight decrease in photosynthetic performance of both *gnat2* and WT plants upon 2 h illumination under 600 μmol photons m^-2^ s^-1^ (19). The same tendency observed in the *gnat2* mutant line revealed that, despite the defects in state transitions (13), the absence of this acetyltransferase does not affect the overall capability of the plants to adjust their metabolism after being transferred to darkness or high light conditions. This observation is in agreement with the findings that state transitions are most important for the acclimation of plants to low light rather than high light conditions (64). In contrast to the 4.5-week-old plants analyzed here, eight-day-old *gnat2* seedlings showed a strong decrease in the maximum quantum yield of PSII after 24 h of constant high light, along with a significant increase in nonregulated photosynthetic quenching (65). Hence, GNAT2 might be important for proper chloroplast development in plants.

High light–induced signals lead to early expression responses, which are followed by slower changes in gene expression. It has been shown that the genes encoding AP2/ERF (APETALA2/ETHYLENE RESPONSE FACTOR) transcription factors are upregulated extremely fast in response to high light (66, 67, 68). Thereafter, genes responding to abiotic stress, especially the family of HSPs, as well as ALTERNATIVE OXIDASE 1a and ascorbate peroxidase APX1 are strongly upregulated (69, 70, 71). The genes regulated by hormones are also enriched among the differentially expressed genes (71).

Under our experimental conditions, the transcriptome analysis after 2 h HL revealed differential transcript expression of approx. 6.5% in WT and 7.5% in *gnat2* plants, respectively. This indicates a profound reprogramming of the transcriptome in both genotypes after the light treatment. There was a substantial overlap of commonly regulated mRNAs, with no antagonistically regulated genes between the two genotypes. This data suggests that the global transcriptome adjustment of *gnat2* upon HL treatment occurred early and followed the same pattern as in the WT. This is different to the *stn7* mutant, which has a defect in state transitions and a similar phenotype as *gnat2*, but is incapable to induce genes related to jasmonate signaling, pathogen defense, and anthocyanin accumulation after 1 h of HL treatment (72). While jasmonate signaling was not induced in our conditions, the transcription factor MYB75, which is important for anthocyanin accumulation upon HL treatment (73), was significantly induced in both WT and *gnat2*. This might indicate that *snt7* has an additional defect in controlling plastid ROS homeostasis (74), which is not the case in *gnat2*. Like in the HL response, similar responses to D were observed with both WT and *gnat2*. It should be noted, however, that transcriptional changes do not necessarily reflect protein abundance shifts, and the photosynthetic capacity of the plant cannot be predicted based on their transcriptome (71, 75, 76, 77, 78). Our quantitative proteomics analysis revealed specific protein accumulations, in dependence of the light treatment or genotype. An accumulation of the HSPs was detected both in WT and *gnat2* in response to the intense illumination (Fig. 3 and Supplemental Data Set 3). While we cannot exclude that a raised leaf temperature from the HL treatment was responsible for the induction of these proteins, recent studies showed that HL itself is probably responsible for the induction of the HSPs (40, 41). In addition, a specific accumulation of PsbTn and TIC62 was observed in *gnat2* compared to WT independent of the light conditions. PsbTn is an interesting candidate protein since it was recently found to play an important role in the acclimation of PSII to light shifts or intense illumination (48). While TIC62 was not detected in our previous study, PsbTn was previously not found to be significantly deregulated in its abundance between WT and *gnat2* (13). Intriguingly, it was recently shown that subtle changes in the growth light conditions affect the growth phenotype of *gnat2* (19), pointing to a possibility that the observed abundance alterations may be dependent on harvest time or microclimate since light intensity, photoperiod, and temperature were similar in both studies.

Successful acclimation of plants to light-induced changes requires not only reprogramming of RNA expression and regulation of protein accumulation but also PTMs of proteins (79). For instance, PSII repair cycle and photosynthetic state transitions are dependent on protein phosphorylation modifications (23, 25, 80, 81). During transition from state 1 to 2, LHCII associates with PSI to balance the excitation energy between the two photosystems. This association is regulated through the light-dependent reversible phosphorylation of the LHCII subunits LHCB1 and LHCB2 (49, 82, 83, 84, 85, 86). We have recently reported that the loss of GNAT2 results in decreased K- and N-terminal acetylation of chloroplast proteins, as well as in defective state transitions under standard light conditions (2, 13). Upon HL treatment, we observed a stronger deregulation of K-acetylation on proteins localized in different subcellular compartments in *gnat2* compared to WT (Fig. 6, *B* and *D*). This might be explained by the action of other acetyltransferases or deacetylases, which compensate for the loss of GNAT2.

Although the high frequency of plastid NTA on subunits of many plastid complexes involved in oxygenic photosynthesis suggested that plastid NTA yield might be dependent on different conditions including light (1), we show here that this does not occur under short-term light treatments. Unlike the N-terminal acetylome, the K-acetylome showed to be very sensitive to the early changes in light conditions especially in the *gnat2* background. This can be explained by the fact that K-acetylation is a reversible PTM that can happen on a faster time scale, while NTA is non-reversible and usually occurs co-translationally or in chloroplasts shortly after protein import and is therefore dependent on protein biosynthesis. In the 2 h time course, hardly any changes in protein abundances were observed. This suggests that K-acetylome marks enable the acclimation in the time regime tested here, while N-terminal acetylation changes might be associated with longer-term responses and/or other signaling responses.

Taken together, our data revealed unique strategies of fast plant acclimation to the different light treatments by lysine acetylation, which suggests that plastid K- and N-terminal acetylations have distinct effects on protein function and therefore mediate different environmental acclimation responses.

## Data Availability

Mass spectrometry proteomics data associated to the N-terminal and lysine acetylomes have been deposited to the ProteomeXchange Consortium (http://proteomecentral.protemeexchange.org).

Lysine acetylome and full proteome data with the identifier JPST001810 can be found at https://repository.jpostdb.org/entry/JPST001810

In addition to that, spectra for acK sites are also accessible *via* MSviewer. The search key for the saved data set is sitefyfimk. The data set can be accessed using the following URL:

https://msviewer.ucsf.edu/prospector/cgi-bin/mssearch.cgi?report_title=MS-Viewer&search_key=sitefyfimk&search_name=msviewer

N-terminomics data are available with the identifier PXD040235.

Transcriptome accession number at Gene Expression Omnibus (GEO; https://www.ncbi.nlm.nih.gov/geo/) is GSE178938.

## Supplemental data

This article contains supplemental data.

## Conflicts of interests

The authors declare that they have no conflicts of interests with the contents of this article.

## Supplementary Data

Supplemental Data Set 1

Supplemental Data Set 2

Supplemental Data Set 3

Supplemental Data Set 4

Supplemental Data Set 5

Supplemental Data Set 6
