# Supplementary material for: Light Changes Promote Distinct Responses of Plastid Protein Acetylation Marks
Source: Mol Cell Proteomics. 2024 Sep 24;23(11):100845. doi: 10.1016/j.mcpro.2024.100845 (PMC11546460; doi:10.1016/j.mcpro.2024.100845)
Supplement: Supplemental Figures 1-8 [file mmc7.pdf]

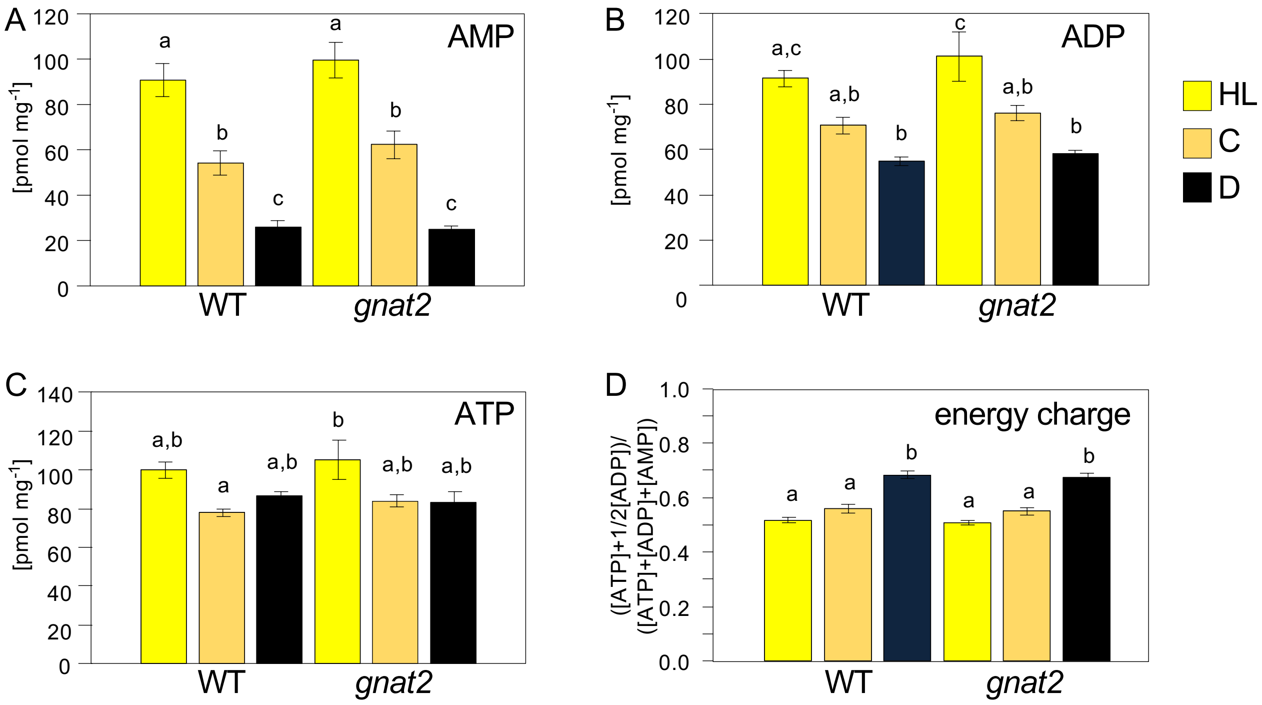

Supplemental Figure 1 - The adenylate status and energy charge in wildtype and *gnat2* after two hours treatment with high light, control light and darkness. Plants were grown on soil under short-day conditions. After 4.5 weeks, the plants were subjected to different light regimes interrupting the short-day light cycle. Two hours after the regular onset of light, the plants were treated with high light illumination (HL, 1000 µmol $m^{-2}s^{-1}$) or darkness (D) for two hours. The control group (Ctrl) continued growth at 120 µmol $m^{-2}s^{-1}$. Rosette material was harvested and the adenylate status (ATP, ADP and AMP) was analysed by metabolite fingerprinting using an ultra-performance liquid chromatography system. The energy charge was computed as ([ATP]+1/2[ADP])/([ATP]+[ADP]+[AMP]). Data given as means ± se. Different letters indicate individual groups identified by pairwise multiple comparisons with a Holm-Sidak, one-way ANOVA ($p < 0.05$, $n = 4$).

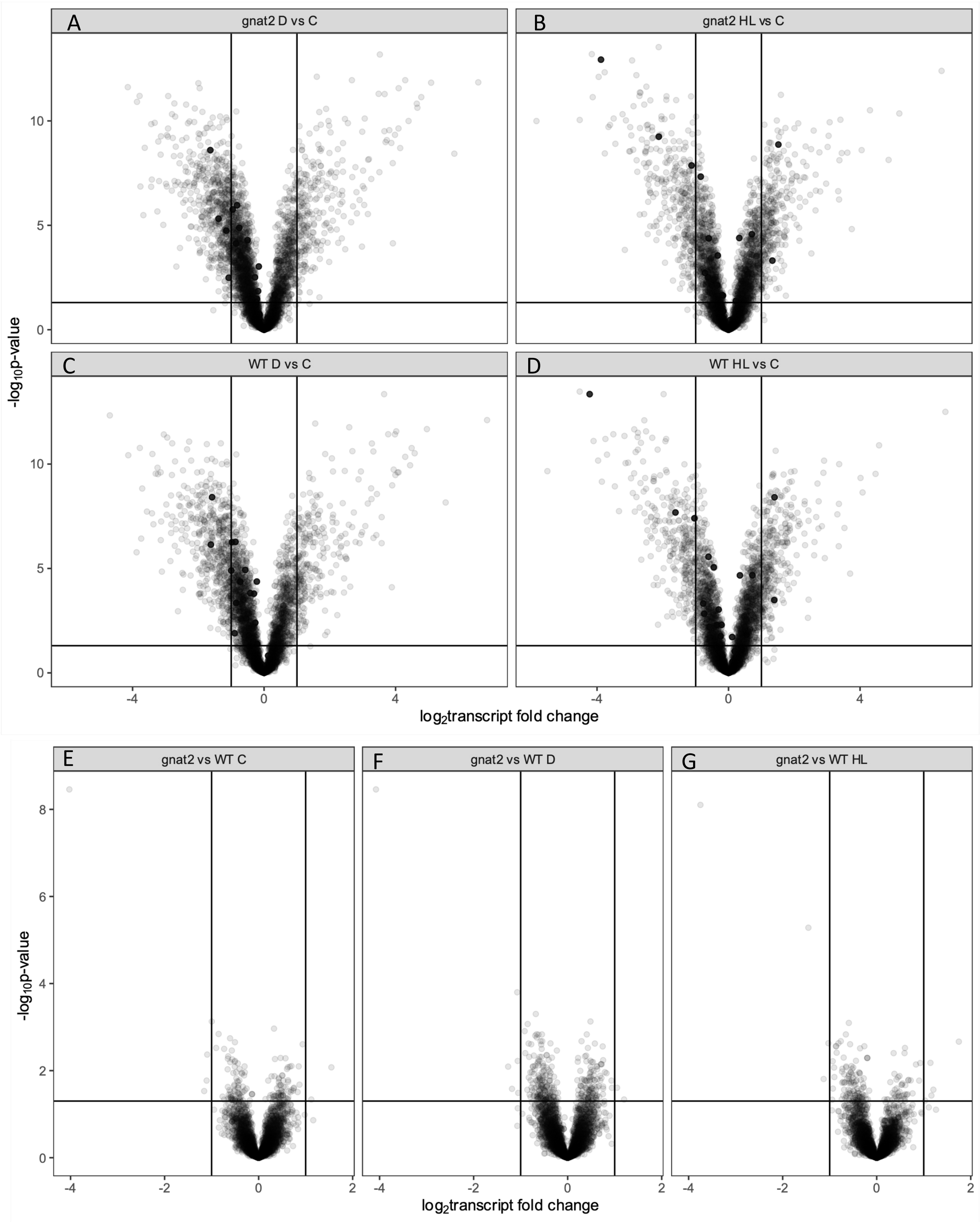

Supplemental Figure 2 - Volcano plots of transcriptome changes. Shown are volcano plots for different light conditions. -$\log_{10}$(p values) are plotted against the $\log_2$ fold-abundance changes +/- 1. LIMMA p value = 0.05 are indicated as solid lines. Opacity is 5 %.

A *gnat2* dark (D) vs control (C)

B *gnat2* high light (HL) vs control (C)

C WT dark (D) vs control (C)

D WT high light (HL) vs control (C)

E *gnat2* vs WT control (C) conditions

F *gnat2* vs WT darkness (D)

G *gnat2* vs WT high light (HL)

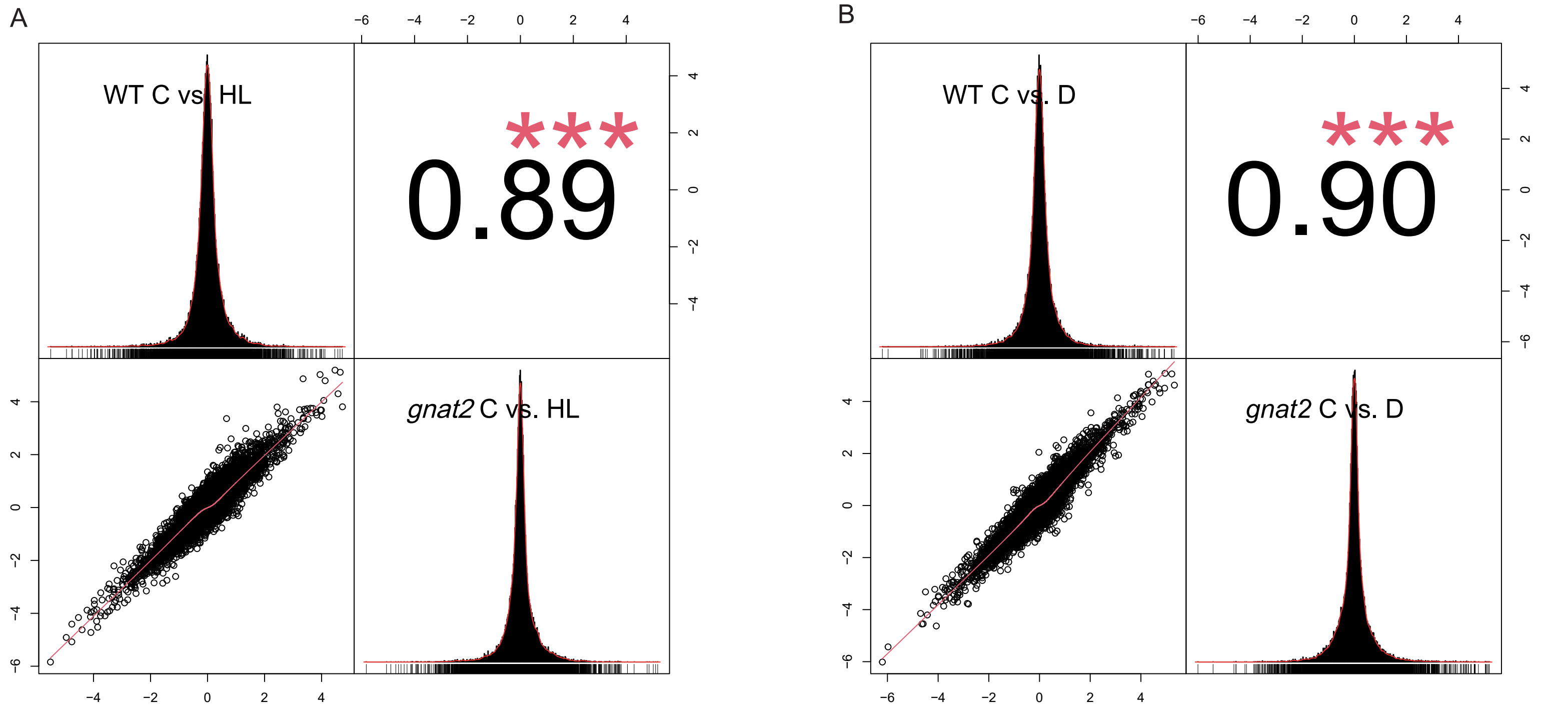

Supplemental Figure 3: multi scatter plots of transcriptome changes. Shown are plots for different genotypes under same light conditions as the $\log_2$ fold-abundance changes and the respective Pearson correlation.

A *gnat2* and WT control (C) vs high light (HL)
B *gnat2* and WT control (C) vs dark (D)

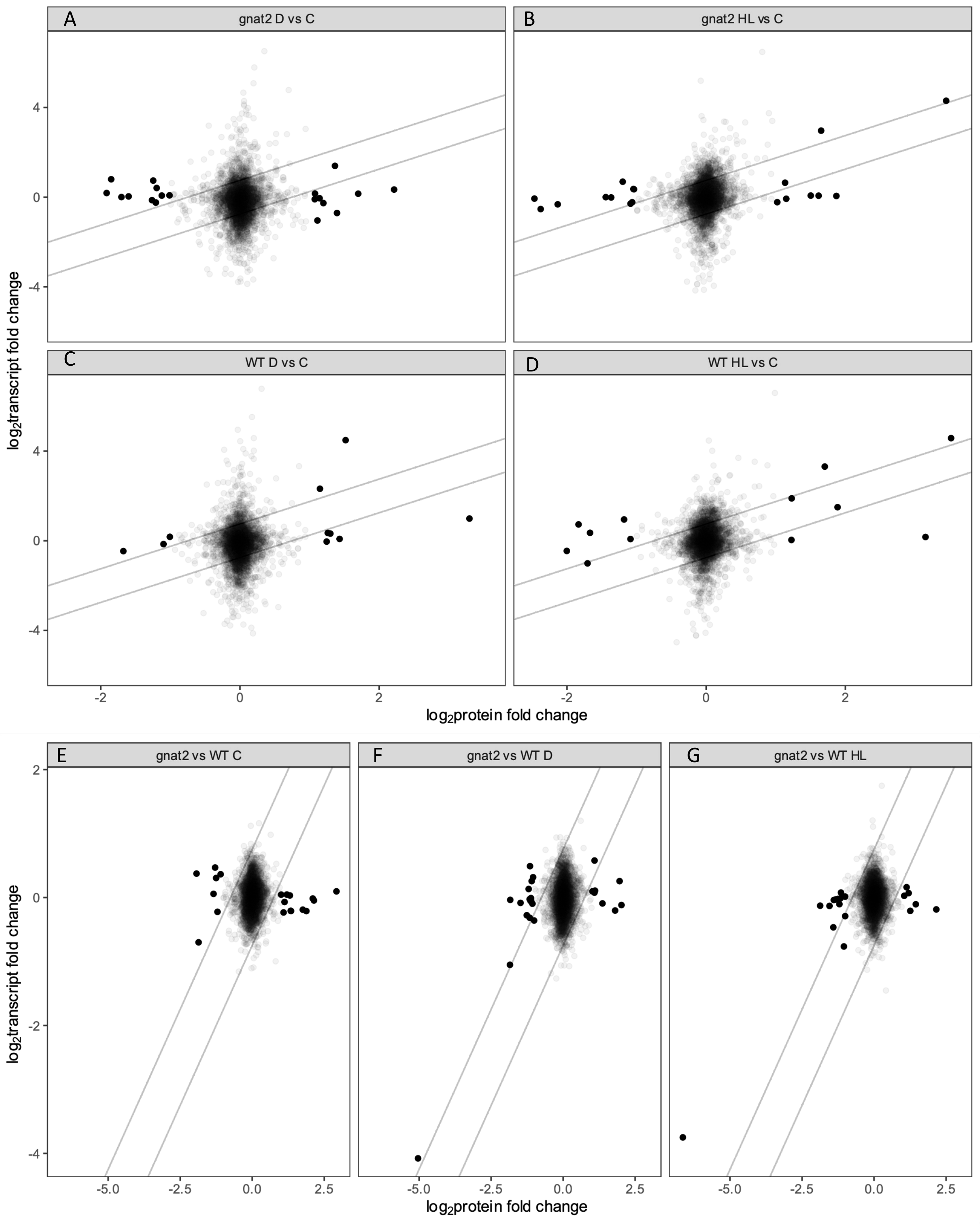

Supplemental Figure 4 - Transcriptome and proteome correlation. Scatter plots showing protein and transcript $\log_2$ fold changes for different light conditions (A+B gnat2 dark (D) and high light (HL), C+D WT D and HL) or genotypes (E-G). Solid reference lines with slope = 1 and intercepts +/- 1 representing the difference in abundance changes determined by either method. Genes with protein log FC > 1 or < -1 indicated as solid dots, otherwise alpha = 0.05.

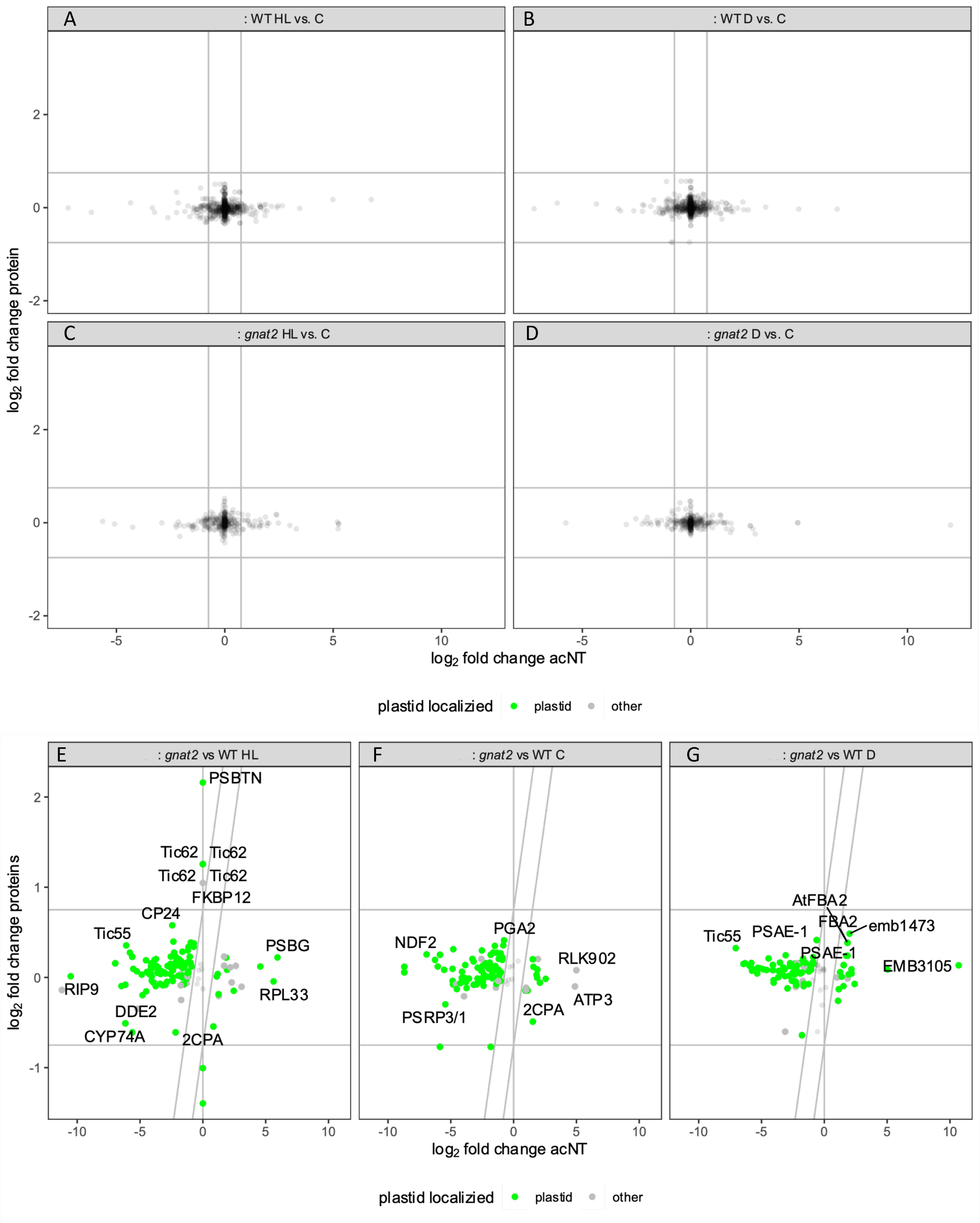

Supplemental Figure 5 - Proteome and NTA correlation.
A-G Scatter plots showing protein and acNT log_2 fold changes for different light conditions (panels A - D) or genotypes (panel E - G). Solid reference lines with slope = 1 and intercepts +/- 0.75 representing the difference in abundance changes between acetylated NT and unmodified proteins carrying the modification, with respective proteins indicated by gene name.

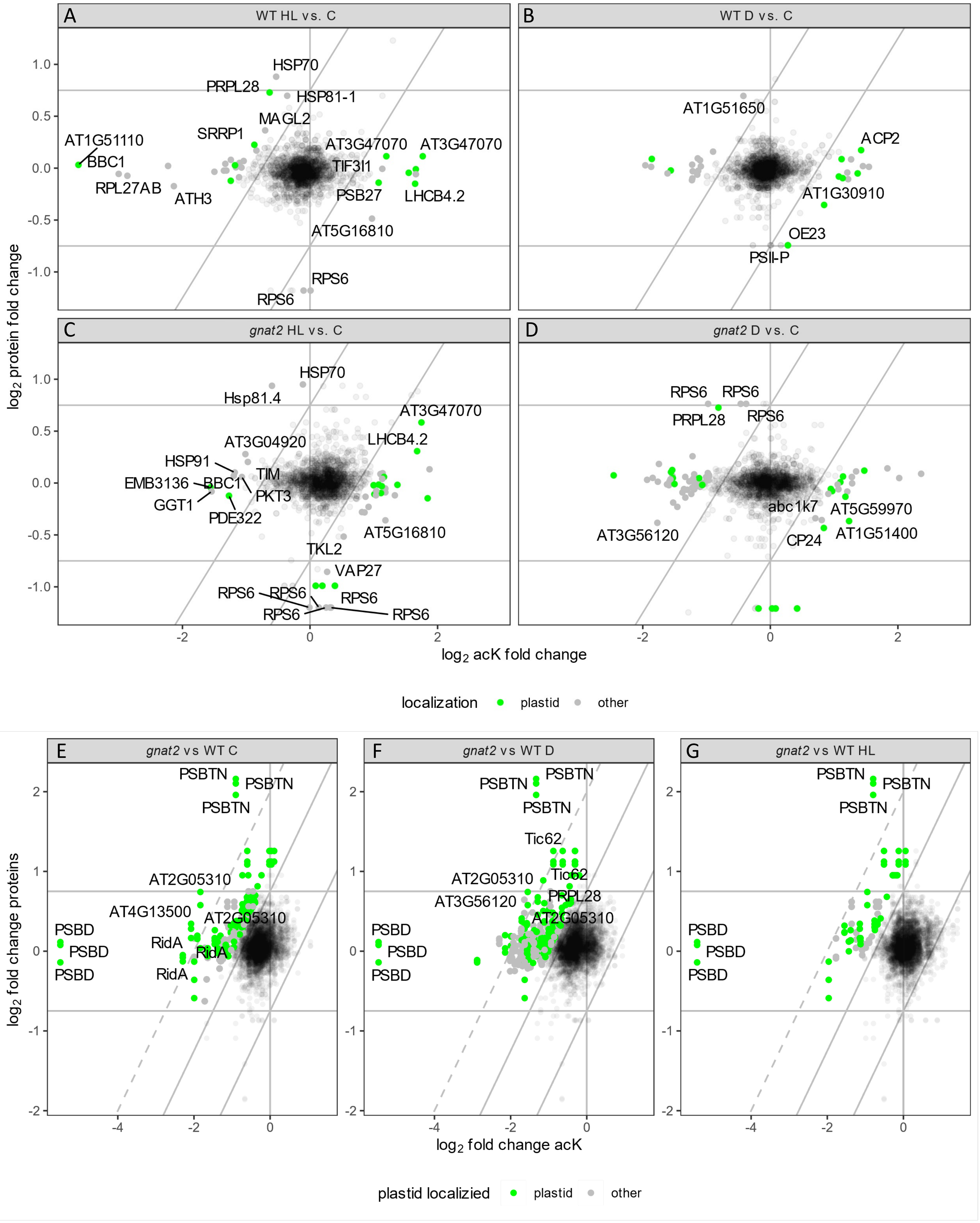

Supplemental Figure 6 - Proteome and acK correlation. Scatter plots showing protein and acK site $\log_2$ fold changes for different light conditions (A-D) or genotypes (E-G). Solid reference lines with slope = 1 and intercepts +/- 0.75 representing the difference in abundance changes between the PTM sites and unmodified proteins carrying the modification, dashed line in panels E-G representing a delta fold change < -2 with respective proteins indicated by gene name.

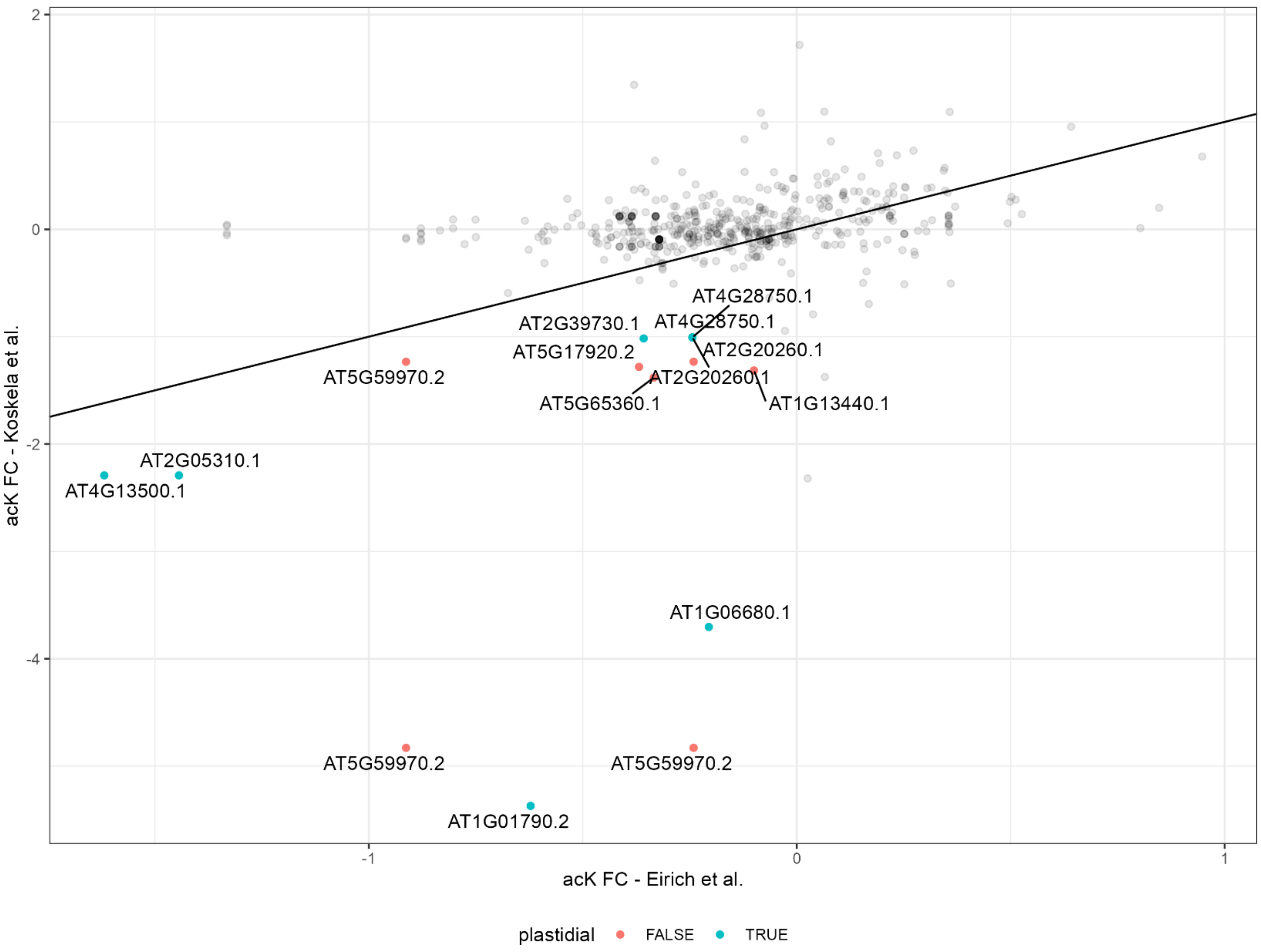

Supplemental Figure 7 - Scatter plot comparing $\log_2$ fold changes of acK sites in WT vs *gnat2* from a previous study by Koskela *et al.* (2018) and the current study. Out of 860 and 2386 sites identified, 622 were matched based on their sequence. Plastidial localication according to SUBcon is given, for a logFC < -1 and 0 respectively the Araport locus is indicated.

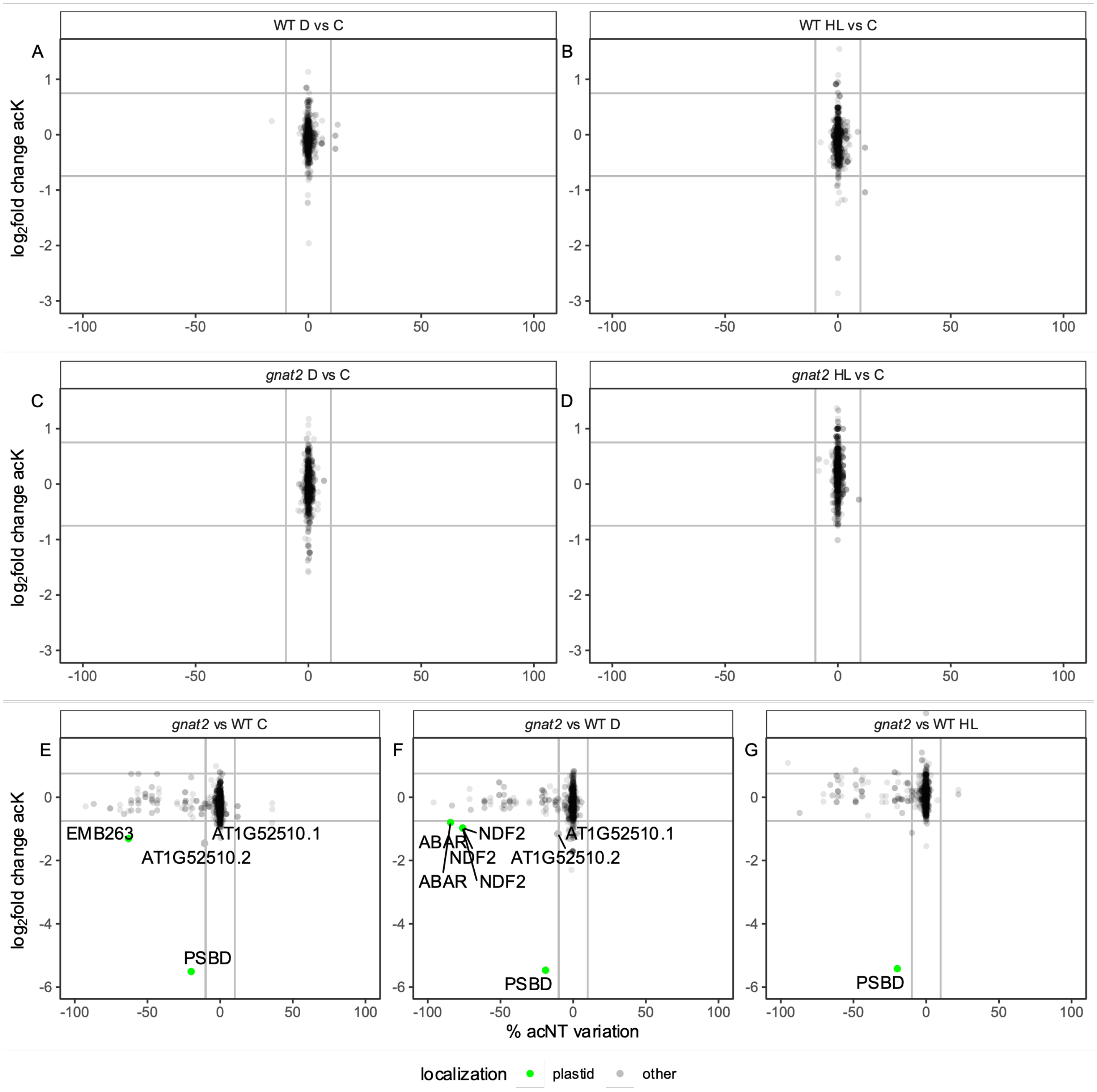

Supplemental Figure 8 - Quantitative comparison of acK and NTA profiling. Scatter plots showing abundance changes of acK and NTA sites (under different light conditions in A-D and per genotype in E-G). $\log_2$ fold-changes +/- 0.75 are indicated as solid lines for acK and NTA variation at +/- 10 %. Plastid localized proteins are indicated in green. Sites with response < -0.75 of acK and < - 10 % to the *gnat2* knock-out are indicated by the respective gene name.
