# Supplementary material for: Light Changes Promote Distinct Responses of Plastid Protein Acetylation Marks
Source: Mol Cell Proteomics. 2024 Sep 24;23(11):100845. doi: 10.1016/j.mcpro.2024.100845 (PMC11546460; doi:10.1016/j.mcpro.2024.100845)
Supplement: Supplemental Table 1 [file mmc8.docx]

**Supplemental Table 1 - Summary of N-terminal profiling of wildtype and *gnat2* after two hours treatment with high light, control light and darkness. This table displays the number of N-termini identified and quantified in the different conditions.** For each sample group, 3 biological replicates were processed as described in Methods. The quantified N-termini were sorted in four different categories: their total number, the ones beginning with the initiating methionine (« iMet »), those that underwent N-terminal methionine excision (« +NME ») and the matured N-termini (« Processed »). In each of these categories, the quantified N-termini were either quantified as fully acetylated (NTA yield > 95%), non-acetylated (NTA < 5%), or partially acetylated.
