# Supplementary material for: Light Changes Promote Distinct Responses of Plastid Protein Acetylation Marks
Source: Mol Cell Proteomics. 2024 Sep 24;23(11):100845. doi: 10.1016/j.mcpro.2024.100845 (PMC11546460; doi:10.1016/j.mcpro.2024.100845)
Supplement: Supplemental Table 3 [file mmc10.docx]

**Supplemental Table 3: Proteins displaying significant NTA decrease (at least 40%) in *gnat2* compared to WT samples, both from the "Dark" condition group.** Entries with a plot number were quantified in both replicates of each condition, allowing the calculation of a p-value; other entries were only quantified in a single replicate of at least one condition.
